# The influence of bioclimate on soil microbial communities of cork oak

**DOI:** 10.1186/s12866-022-02574-2

**Published:** 2022-06-23

**Authors:** Daniela Costa, Rui M. Tavares, Paula Baptista, Teresa Lino-Neto

**Affiliations:** 1 Centre of Molecular and Environmental Biology (CBMA), Department of Biology, University of Minho, Campus of Gualtar, 4710-057 Braga, Portugal; 2 Centro de Investigação de Montanha (CIMO), Instituto Politécnico de Bragança, Campus de Santa Apolónia, 5300-253 Bragança, Portugal

**Keywords:** Soil microbiome, ITS, 16S, Cork oak, Bioclimate

## Abstract

**Background:**

Soil microbiomes are important to maintain soil processes in forests and confer protection to plants against abiotic and biotic stresses. These microbiomes can be affected by environmental changes. In this work, soil microbial communities from different cork oak Portuguese forests under different edaphoclimatic conditions were described by using a metabarcoding strategy targeting *ITS2* and *16S* barcodes.

**Results:**

A total of 11,974 fungal and 12,010 bacterial amplicon sequence variants (ASVs) were obtained, revealing rich and diverse microbial communities associated with different cork oak forests. Bioclimate was described as the major factor influencing variability in these communities (or bioclimates/cork oak forest for fungal community), followed by boron and granulometry. Also, pH explained variation of fungal communities, while C:N ratio contributed to bacterial variation. Fungal and bacterial biomarker genera for specific bioclimates were described. Their co-occurrence network revealed the existence of a complex and delicate balance among microbial communities.

**Conclusions:**

The findings revealed that bacterial communities are more likely to be affected by different edaphoclimatic conditions than fungal communities, also predicting a higher impact of climate change on bacterial communities. The integration of cork oak fungal and bacterial microbiota under different bioclimates could be further explored to provide information about useful interactions for increasing cork oak forest sustainability in a world subject to climate changes.

**Supplementary Information:**

The online version contains supplementary material available at 10.1186/s12866-022-02574-2.

## Background

Soil microbial communities are affected by environmental changes [1] and remain affected even after the end of extreme events, like drought [2]. Besides weakening plants, such extreme events have serious impacts on the structure of microbial communities [3], including the increase on the relative abundance of rare species [2] and emergence of pathogens able to infect plants [4]. On the other hand, stressed plants are able to recruit specific rhizosphere microbes, by changing the composition of their root exudates, thus modulating their adaptation and survival to such adverse conditions [5, 6]. Rhizosphere soil microbiome is composed by different microorganisms, including fungi and bacteria, which live in a complex environment [7] and respond differently to environmental changes [8–11]. Such microorganisms can establish symbiotic relationships with plants [12, 13] and interact with each other for influencing the biotic and abiotic environment [14]. For example, the interaction between mycorrhizal fungi and mycorrhiza helper bacteria (MHB) can increase root colonization and mycorrhiza formation, reducing plant stress [15, 16] and enhancing mineralization of organic phosphorus on soil [17]. Also, bacteria are important for the development of ectomycorrhizal fruit bodies [18]. The ecological services played by microbial soil communities, including plant growth promotion, stress tolerance, disease resistance, and their participation on biogeochemical cycles (mainly carbon, nitrogen and phosphorous) [19], turns the study of such soil communities an important issue for understanding climate change outcomes.

Climate models are showing that the Mediterranean region is a climate change hotspot [20]. The combined effects of decreasing precipitation and increasing temperature place Mediterranean forests as one of the most vulnerable ecosystems [21]. Among these, cork oak (*Quercus suber* L.) forests cover a large area of the western Mediterranean, having a high ecological and socio-economic importance [22]. The impact of climate changes on these forests have fostered cork oaks decline, which has been increasingly reported within the Mediterranean region [23]. Cork oak health is expected to be even more affected by drought events due to the emergence and spread of pathogens resulting from climate changes [23]. As generally recognized for woody plants [24], the microbial communities residing in cork oak forests could play a crucial role for preserving this ecosystem biodiversity and functionality. In soils of cork oak forest, the fungal communities have been better described than bacterial ones. Most studies relied on the identification of fungal communities using fruiting bodies surveys (e.g. [25]), culture-based methods (e.g. [26]) or root tips barcoding (e.g. [27, 28]). Fewer studies have used metabarcoding approaches for studying fungal communities associated with cork oak forests and the existent were focused on the fungal diversity found in different land-uses of these forests [29, 30]. For studying the impact of land management intensity on soil microbial diversity, a single study reported the simultaneous survey of bacterial and fungal microbiota by using a high throughput sequencing approach [31]. The integration of data obtained from fungal and bacterial microbiota is of utmost importance for understanding the functioning of any ecosystem and could provide important clues for the conservation of soil health, even when considering climate change scenarios [32]. Taking this into account, using a metabarcoding approach we aim to understand: (i) the divergence in microbial (bacteria and fungi) composition of cork oak forests located in regions with different bioclimates (combination of precipitation and temperature data), (ii) which edaphoclimatic factors have more influence in shaping these communities, and (iii) the correlation between fungal and bacterial biomarkers in different bioclimates. Final outcomes are expected to provide new information for a better management of cork oak forests under predicted climate changes.

## Material and methods

### Sampled forests

Soil samples were collected from eight cork oak Portuguese forests with different bioclimate classifications (Table S1; Fig. S1). Bioclimate classification was performed using Emberger climatogram and Emberger index *Q* = (2000 x *P*)/(*M*^2^ - *m*^2^), where *P* is the annual precipitation (mm), *M* is the maximum temperature in the warmest month (K) and *m* is the minimum temperature in the coldest month (K) [33–35]. Precipitation and temperature data spanning 10 years (2006–2016) previous to sampling were extracted from TerraClimate dataset [36], using QGIS [37] with a 0.05 buffer for each location. Sampled forests also presented different forest systems [*sobreiral* (about 400 trees/ha) or *montado* (60–100 trees/ha)], use of forest (wild forest or forest pasture) and tillage (tilled or non-tilled).

### Cork oak soil samples

From each forest, cork oak trees [6 in Limãos (LI), Alcobaça (AL), Gavião (GV), Grândola (GR), Herdade da Contenda (HC-CT and HC-MA); and 5 in Parque da Peneda-Gerês (PG-ER and PG-RC)] were randomly selected and three equidistant soil core samples were collected from the middle of each tree canopy, at 2–10 cm in depth, in 2017 (Table S1). Soil samples were stored in sterile plastic bags at 4 °C, until processing. For physicochemical analyses, soil samples from each forest were thoroughly mixed and sent to a service provider (A2 - Análises Químicas, Portugal). Analyses comprised granulometry, pH (H_2_O and CaCl_2_), electric conductivity (μS/cm), organic matter, organic carbon, total nitrogen, carbon:nitrogen ratio and elements [phosphorus (P_2_O_5_), potassium (K_2_O), calcium (CaO), magnesium (MgO), sulfur, iron, manganese, boron, and sodium] (Table S2). The remaining soil was sieved (45-mesh sieve) to remove root tissues and other larger components of soil, thoroughly homogenized and three soil replicates per forest were created and stored at − 80 °C.

### DNA samples preparation and Illumina sequencing of soil microbes

DNA extraction was performed for each soil replicate using *DNeasy PowerSoil Kit* (Qiagen, Germany), according to manufacturer recommendations. DNA amplification was assessed by PCR assays using a) *ITS1-F* (CTTGGTCATTTAGAGGAAGTAA, [38]) and *ITS2* (GCTGCGTTCTTCATCGATGC, [38]) primers for the internal transcribed spacer 1 (*ITS1*) region of fungi, and b) *799F* (AACMGGATTAGATACCCKG, [39]) and *1492R* (GGTTACCTTGTTACGACTT, [39]) primers for the *16S* V5-V9 bacterial region. The PCR reaction mixtures (25 μl) contained 1x Complete NH_4_ reaction buffer (Bioron GmbH, Germany), 200 μM of each dNTP (NZYTech, Portugal), 5 μM of each primer, 1.25 U DFS-*Taq* DNA Polymerase (Bioron GmbH, Germany) and 1 μl of DNA template (20 ng/μl). Amplifications were performed in a T100™ Thermal Cycler (Bio-Rad, USA), using the following protocol: initial denaturation for 4 min at 94 °C; 35 cycles of 30 s at 94 °C, 30 s at 52 °C (*ITS1*) or 54 °C (*16S*) and 30 s at 72 °C; final elongation at 72 °C for 10 min. PCR products were run on a 1% (w/v) agarose gel, stained with Green Safe Premium (NZYTech, Portugal). DNA samples that resulted in amplification were quantified using a fluorescent DNA quantification assay with *dsDNA HS Assay Kit* (ThermoFisher Scientific, USA) and detected with a Qubit 3.0 Fluorometer (ThermoFisher Scientific, USA).

DNA samples presenting a concentration higher than 20 ng/μl (three samples/forest) were sequenced using an *Illumina MiSeq®* sequencer with the V3 chemistry*,* through paired-end sequencing (2 × 300 bp) by a service provider (Genoinseq, Portugal). To determine fungal community, a first PCR reaction was performed to target hypervariable region of *ITS2* using a pool of forward primers [*ITS3NGS1_F* (CATCGATGAAGAACGCAG), *ITS3NGS2_F* (CAACGATGAAGAACGCAG-3), *ITS3NGS3_F* (CACCGATGAAGAACGCAG), *ITS3NGS4_F* (CATCGATGAAGAACGTAG), *ITS3NGS5_F* (CATCGATGAAGAACGTGG-3), and *ITS3NGS10_F* (CATCGATGAAGAACGCTG)] and the reverse primer *ITS4NGS001_R* (TCCTSCGCTTATTGATATGC) [40]. Separately, to determine bacterial community a first PCR reaction was performed to target hypervariable *V5-V7* region of *16S* using the forward primer *799F-Y* (AACMGGATTAGATACCCKG) and the reverse primer *1193R* (ACGTCATCCCCACCTTCC) [39, 41]. A second PCR reaction, for each community, added indexes and sequencing adapters to both ends of the amplified target region, according to manufacturer’s recommendations [42].

### Reads processing and data analysis

Raw reads were extracted from *Illumina MiSeq*® System in fastq format. Sequencing adapters and reads with less than 100 bases were removed with *PRINSEQ* version 0.20.4 [43]. Trimming, based on quality scores, was performed using default parameters in *Sickle* [44]. Before merging, correction of errors in reads was performed using *Bayeshammer* module from *SPAdes* package [45]. The merging of overlapping paired-end reads (Merged reads; Table S3) and further quality filtering was performed using *Usearch* version 8.0.1623 [46]. *Fastq-mcf* from *ea-utils* package [47] was used to filter merged reads based on sequence size. The software *micca* version 1.7.0 [48] was used to create a single FASTQ file (*micca merge*) and to discard sequences with an expected error rate greater than 1% (*micca filter*). This software (*micca otu)* was also used to cluster amplicon sequence variants (ASV), by using UNOISE3 protocol and chimeric sequences removal (Reads clustered into ASV; Table S3). Taxonomy was assigned to each ASV using a reference database [UNITE database version 8.2 [49] for fungi and *qiime*-compatible Silva release 132 [50] for bacteria] with *micca classify*. ASV assigned as unclassified were removed from bacterial dataset. In fungal dataset, those ASV assigned as unclassified, Viridiplantae, Algae or others not corresponding to fungal ASV were removed. Biom-format tables were created, and each dataset was subsampled using *Qiime* version 1.9.0 [51] to the number of sequences in the sample with the lowest number (Table S3; fungi to 26,787 that was found in GR1; bacteria to 25,087 sequences that was found in AL3).

### Diversity and statistical analysis

All analyses were performed using the subsampled datasets in *RStudio* version 4.0.2 [52]^,^ except when stated otherwise. Fungal and bacterial richness (*S*) and diversity [Gini-Simpson’s index (1-*D*) and Shannon’s index (*H*′)] were determined for the different groups of samples using *alpha()* function of *microbiome* package [53]. While 1-*D* index measures the evenness of a community and represents the probability that two individuals randomly selected from a sample will belong to different species [54, 55], *H*′ index measures the diversity by taking into account the number of individuals as well as the number of taxa [54, 56]. These ecological parameters were compared between samples from different bioclimates, where hyper-humid comprised results from PG-ER and PG-RC forests, humid from LI and AL forests, sub-humid from GV and GR forests, and semi-arid from HC-CT and HC-MA forests. Statistical analysis were performed using *stat_compare_means()* from *ggpubr* package [57]. Rarefaction curves were computed to determine sampling effort using *rarecurve()* of *vegan* package. Fungal and bacterial abundance and richness were determined for the different bioclimates using *microbiome* [53] and *phyloseq* [58] packages.

Non-metric multidimensional scaling (NMDS) was performed using square root transformation of data for all samples to understand community distribution. NMDS was performed using *vegdist()* of *vegan* package [59] to calculate Bray-Curtis dissimilarity indices matrix, *envfit()* to understand goodness of fit (999 permutations) of environmental variables and *metaMDS*() of the same package to obtain the ordination graph. Goodness of fitness of the model was measured using Kruskal’s stress (values lower than 0.2 represent a good ordination; [60]. For evaluating differences on microbial communities from distinct bioclimates, ANOSIM was performed in *Community Analysis Package* 5.0 [61], using the Bray-Curtis dissimilarity matrix. This matrix was also used to perform multivariate statistical tests of significance (PERMANOVA), by using *adonis()* function of *vegan* package [59] with 999 permutations and homogeneity of group dispersions (PERMDISP). To determine which edaphoclimatic factors were more important in shaping fungal and bacterial communities a redundancy analysis (RDA) was performed using the package *vegan* [59]. Trend surface analysis was performed to transform latitude-longitude data into flat Cartesian coordinates using *geoXY()* of SoDA package [62] and polynomials of degree 3 were computed using *poly()* of STATS package [52]. Flat Cartesian coordinates (designated as cork oak forest) were included in RDA with other edaphoclimatic variables (bioclimate, forest system, tillage, and soil parameters). The function *rda()* was used to perform redundancy analysis, while *anova.cca()* was used to perform Monte Carlo permutation test (1000 permutations) and to evaluate the significance of global model. Most parsimonious model was found by forward selection of explanatory variables using *ordistep()* with 999 permutations. Multicollinearity of variables was evaluated using *vif.cca()* and excluded if variance inflation factors (vif) > 20. The variation explained by the model was tested using *RsquareAdj()* and Monte Carlo permutation test (1000 permutations) to evaluate significance. To understand the contribution of each variable for the variation of microbial community, variation partitioning was performed for the most parsimonious model using *varpart()* function. Statistical significance of variables and conditional variables was assessed using *test_vp4()* from *comecol* package [63].

The remaining analyses were performed using only ASVs classified up to genus level. The fungal and bacterial core community were related with bioclimate classification using *heatmap.2()* function of *gplots* package [64]. Linear discriminant analysis Effect Size (LEfSe) was used (LDA score > 4; *p* < 0.05) to discover bioclimate biomarkers [65], which consist in those microbial genera that could explain differences in bioclimate (the most significant environmental factor revealed by RDA). An adjacent matrix was created using *Hmisc* [66] and *Matrix* [67] packages, to perform a co-occurrence network of biomarker genera considering Pearson’s correlation coefficient higher than 0.75 (*p* < 0.05). Adjacent matrix was open in Gephi version 0.9.2 [68] to visualize co-occurrence network. The functional groups of biomarker fungal and bacterial genera were assessed using *FunGuild* [69] and *FAPROTAX version 1.2.4* [70], respectively. For correlating biomarker genera and functional groups, correlation matrixes were computed using Pearson’s correlation coefficient with *rcorr()* function of *Hmisc* package [66]. *Corrplot()* function from *corrplot* package [71] was used to visualize correlation matrixes, by only presenting statistically significant correlations (*p* < 0.05).

## Results and discussion

### Fungal and bacterial dataset processing

Fungal and bacterial communities of soil from cork oak forests with different bioclimates were analysed by pair-end *Illumina MiSeq* platform, using *ITS2* and *16S* barcodes, respectively. Using *ITS2* as a barcode, a total of 2,365,263 raw reads were obtained from 24 cork oak soil samples, ranging from 40,904 to 172,166 raw reads per sample (Table S3). Processed reads (1958803) contained around 3.2% of non-fungal ASVs, including 1.9% unclassified sequences, 1.3% Viridiplantae, 0.03% Alveolata and 0.008% Metazoa reads. Sequences corresponding to these ASVs were removed, resulting in 1,897,062 high quality fungal sequences, which were clustered into 11,997 ASVs (amplicon around 400 bp). To mitigate potential bias introduced by different sequencing depth in each sample, dataset subsampling was performed to the sample with least number of fungal sequences (GR1; 26,787 sequences). After subsampling, a total of 642,888 sequences were clustered into 11,974 fungal ASVs (Table S4A), which were assigned to 15 phyla, 37 classes, 91 orders, 170 families, 302 genera and 178 species. The same methodology was used for the bacterial dataset. From all cork oak soil samples (24), a total of 1,610,298 raw reads were obtained by using *V5-V7* region of *16S* region as a barcode, ranging from 50,673 to 91,233 raw reads per sample (Table S3). Processed reads (819012) contained 0.1% non-bacterial sequences, all of which corresponding to unclassified ASVs. After non-bacterial ASVs removal, a total of 818,092 high-quality bacterial sequences were obtained, which were clustered into 12,035 ASVs (amplicon around 400 bp). Bacterial dataset subsampling was performed to the sample with the least number of bacterial sequences (AL3; 25,087 sequences). After subsampling, a total of 602,088 sequences were clustered into 12,010 bacterial ASVs (Table S4B), which were assigned to 22 phyla, 53 classes, 107 orders, 142 families, 248 genera and 16 species. Although in this work the percentage of unclassified microorganisms was low (1.9 and 0.1%, when using *ITS2* and *16S* barcodes, respectively), there were many ASVs that did not match lower taxonomic levels, such as genus (Fungi 60%; Bacteria 46.9%) or species (Fungi 92%; Bacteria 99.7%). This result supports the assumption that much of the microbial diversity present in soils still remains to be discovered and studied [72, 73].

### Cork oak forests microbial communities: diversity and composition

**Fig. 1:**
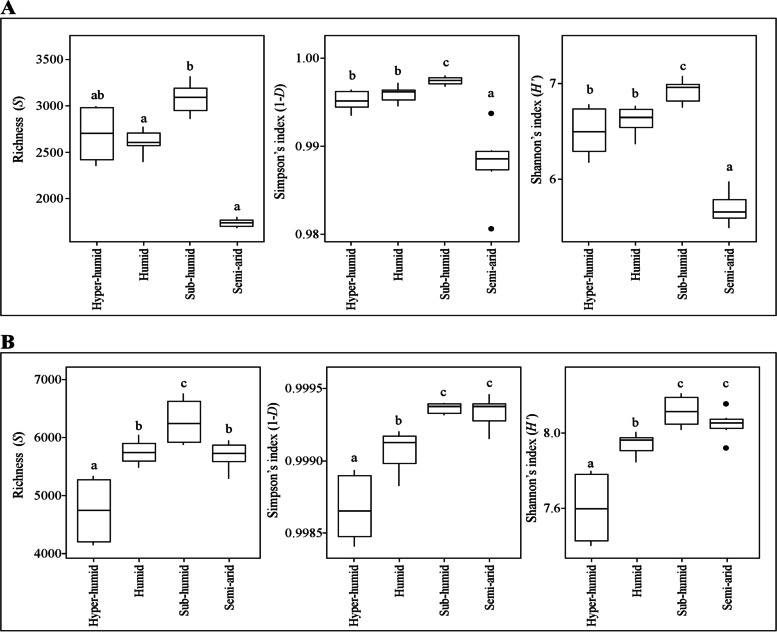
Diversity of fungal (**A**) and bacterial (**B**) communities found in cork oak forest soils from each bioclimate. Each bioclimate is represented by two different forests [hyper-humid comprising PG-ER and PG-RC forests (*n* = 6); humid, LI and AL forests (*n* = 6); sub-humid, GV and GR forests (*n* = 6); semi-arid, HC-CT and HC-MA forests (*n* = 6)]. *S* represents microbial richness, 1-*D* Gini-Simpson’s index and *H*′ Shannon’s index. Different letters mean statistically significant differences, determined by ANOVA followed by Kruskal-Wallis test (*p* ≤ 0.05)

Microbial communities varied in richness and diversity in cork oak soils from different forests (Fig. S2) and bioclimates (Fig. 1). Rarefaction curves agreed with these results and revealed the good representation of microbial communities, especially when considering all soil samples from a certain bioclimate (Fig. S3). Although rarefaction curves for different individual forests did not reach a plateau (Fig. S3A, C), the sampling effort was good enough to represent microbial communities from different bioclimates (Fig. S3B, D). Taking this into account, and due to the meaningful ecological outcomes, results will be always discussed considering bioclimates. Soils taken from sub-humid bioclimates always presented the richest and more diverse microbial assemblages, both for bacterial and fungal communities (Fig. 1). Interestingly, semi-arid soils were particularly poor in fungal taxa and diversity (Fig. 1A) but were rich and diverse concerning bacterial communities (Fig. 1B). This result agrees with previous studies that described higher bacterial richness and abundance on soils subjected to drought [74, 75]. Such results have been explained by the theory of low pore connectivity, in which water-filled soil pores are poorly connected to each other in dried soils, affecting competitive interactions between bacterial species, which result in an increased bacterial diversity [74]. Previous studies also revealed the influence of climate on fungal communities [76]. In the present work, the most humid places were the ones that displayed higher fungal diversity and richness compared to semi-arid forests. This result could arise from the facilitation of fungal development under high relative humidity [77, 78]. Furthermore, this result is in line with the diversity found in ectomycorrhizae (ECM) root tips from cork oak stands [28], suggesting that was the higher diversity found in soils that contributed to the increased number of ECM root tips and not the promotion of mycorrhization process by itself.

**Fig. 2:**
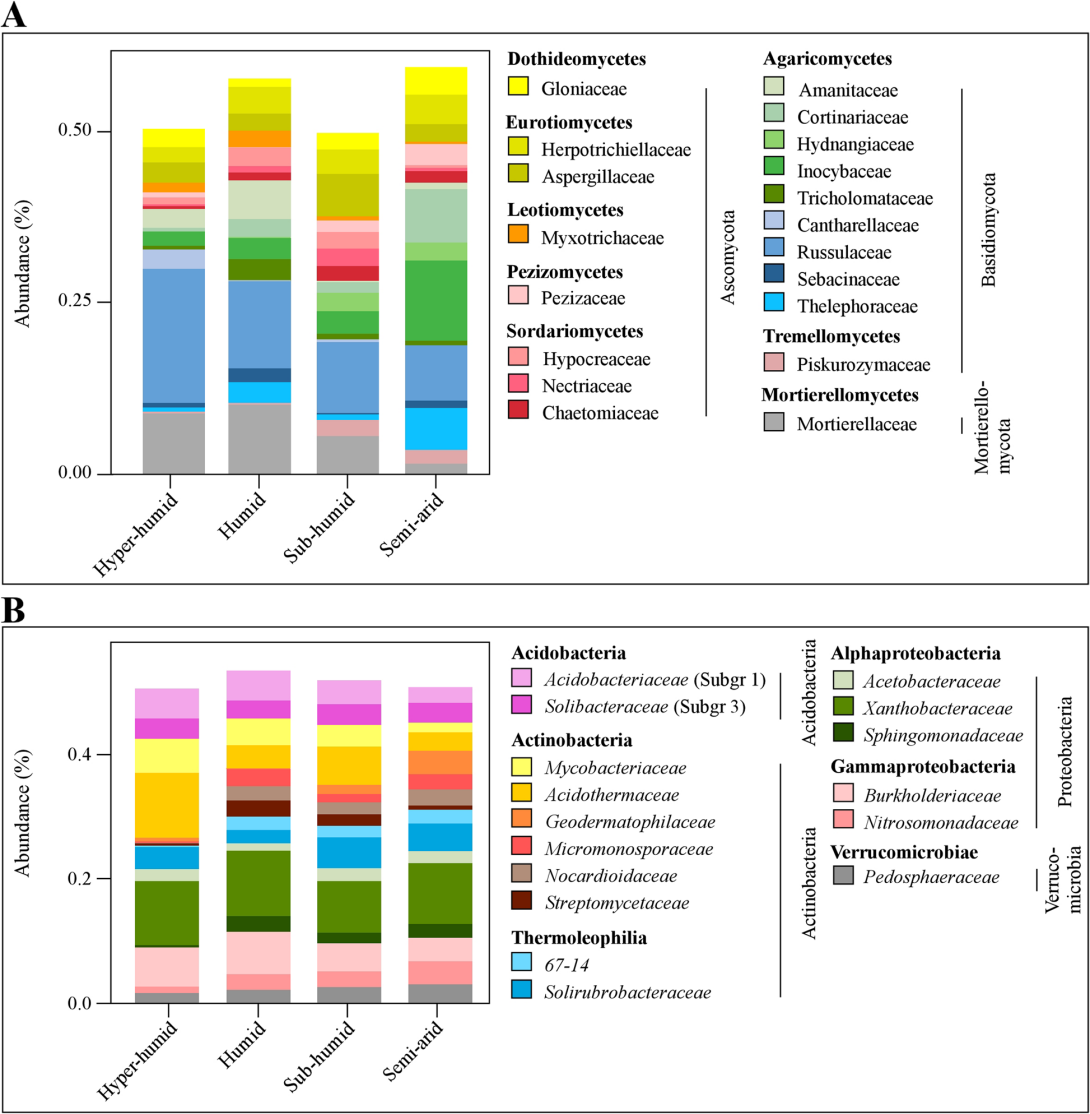
Mean relative abundance of soil fungal (**A**) and bacterial (**B**) communities in forests from each bioclimate. Results display communities at family level, being indicated the corresponding classes and phyla

The dominant phyla in fungal community were Ascomycota and Basidiomycota, representing 41 and 22% of richness and 34 and 39% of abundance, respectively (Fig. S4). The prevalence of these phyla agrees with other studies in cork oak [29–31]. Agaricomycetes (21% richness; 37% abundance), Eurotiomycetes (10% each) and Sordariomycetes (9% each) were the richest and most abundant classes. Agaricomycetes was mostly represented by Agaricales (9% richness, 16% abundance) and Russulales (4% richness, 13% abundance). As expected from a Fagaceae forest [79], ectomycorrhizal species dominated in diversity (18% of richness), but particularly in abundance (37%), mainly regarding the fungal families Russulaceae (13% of total abundance), Inocybaceae (5%), Cortinariaceae (3%) and Thelephoraceae (3%). These results are in line with other fungal metabarcoding studies on Fagaceae forests that also described an enriched ECM Basidiomycota community [80, 81]. Besides, these ECM families were considered as ubiquitous in Mediterranean cork oak forests [82]. When comparing the composition of soil forest fungal assemblages among different bioclimates, a similar richness profile was found (Fig. S5A), but differences in fungal abundance were detected in different bioclimates (Fig. 2A). More humid forests were particularly enriched in Russulaceae [ECM, 16.2% in most humid (hyper-humid and humid) forests against 9.2% in driest (sub-humid and semi-arid) ones] and Mortierellaceae (non-ECM, 9.6% against 3.6%), but also displayed high levels of Amanitaceae (ECM, 4.5% against 0.6%) and Cantharellaceae (ECM, 1.5% against 0.2%). The opposite pattern was observed for Inocybaceae (ECM, 2.7% against 7.6%), Cortinariaceae (ECM, 1.6% against 4.8%), and Hydnangiaceae (ECM, 0.4% against 3%) families. The same family distribution trend was observed for ECM root tips [28], suggesting once more the relation of ECM fungal abundance and mycorrhization of cork oaks. Other non-ECM families also displayed higher abundance in less humid bioclimates, like Piskurozymaceae family (0.2% in most humid forests against 2% in driest ones). But the most noteworthy difference of non-ECM fungi among different bioclimates was the higher occurrence of the abundant Mortierellaceae family in humid and sub-humid forests. Besides being humid, these forests are characterized by lower annual temperatures throughout the year, which could favour the development of these fungi, as warmed soils were recently reported to reduce the abundance of Mortierellaceae members [83]. As the most humid forests are mostly wild forests with high density of trees, the prevalence of Mortierellaceae members in these forests could be also related with their ecological function as saprophytic fungi, decomposing forest litter through chitin and lignin degradation [84]. However, bioclimate seems to be more important for Mortierellaceae abundance (PERMANOVA: *R*^2^ = 0.66, *p* = 0.001) than forest use (PERMANOVA: *R*^2^ = 0.25, *p* = 0.001).

Regarding bacterial community, *Proteobacteria*, *Actinobacteria* and *Acidobacteria* were the most prevalent phyla with 35, 29 and 19% richness and 37, 32 and 18% abundance, respectively (Fig. S6). Similar profiles have been detected in other forest soils (e.g., [85]) and cork oak forests ([75]). The richest and more abundant classes were *Alphaproteobacteria* (19% richness; 22% abundance), *Actinobacteria* (17%; 21%), *Acidobacteriia* (16%; 17%) and *Gammaproteobacteria* (12%; 13%). Of relevance was the dominance of *Xanthobacteraceae* (*Alphaproteobacteria*, 10% of total abundance), followed by *Acidothermaceae* (*Actinobacteria*, 6%) and *Burkholderiaceae* (*Gammaproteobacteria*, 5%) families, all including well-known members able to interact with plants. For example, *Xanthobacteraceae* comprise species with plant growth promotion abilities [86] and nitrogen fixation in legumes [87]. The highest prevalence of *Acidothermus* sp. (*Acidothermaceae*) and *Burkholderia* sp. (*Burkholderiaceae*) have been described in different forests, including cork oak forests [75, 88]. As detected for fungal communities, a similar profile of richness was found in cork oak forests from different bioclimates (Fig. S5B). Regarding taxa abundance, differences between bioclimates were perceived, but not as clearly as in fungal community (Fig. 2B). Besides presenting a general high abundance, *Acidothermaceae* and *Burkholderiaceae*, as well as *Mycobacteriaceae* were more abundant in the most humid forests (4.2, 6.6 and 4.8%, respectively) than in the drier ones (2.6, 4.2 and 2.6%, respectively). The opposite pattern was shown for *Solirubrobacteraceae* (2.9% against 4.8%) and *Nitrosomonadaceae* (1.8% against 3.1%). The most arid bioclimates also seem to favour bacterial proliferation of *Geodermatophilaceae* (0.4% in most humid against 3% in the driest forests). Taken together, these results suggest a bioclimate impact on the abundance of some microbial taxonomic groups.

### Environmental factors influencing microbial community structure

**Fig. 3:**
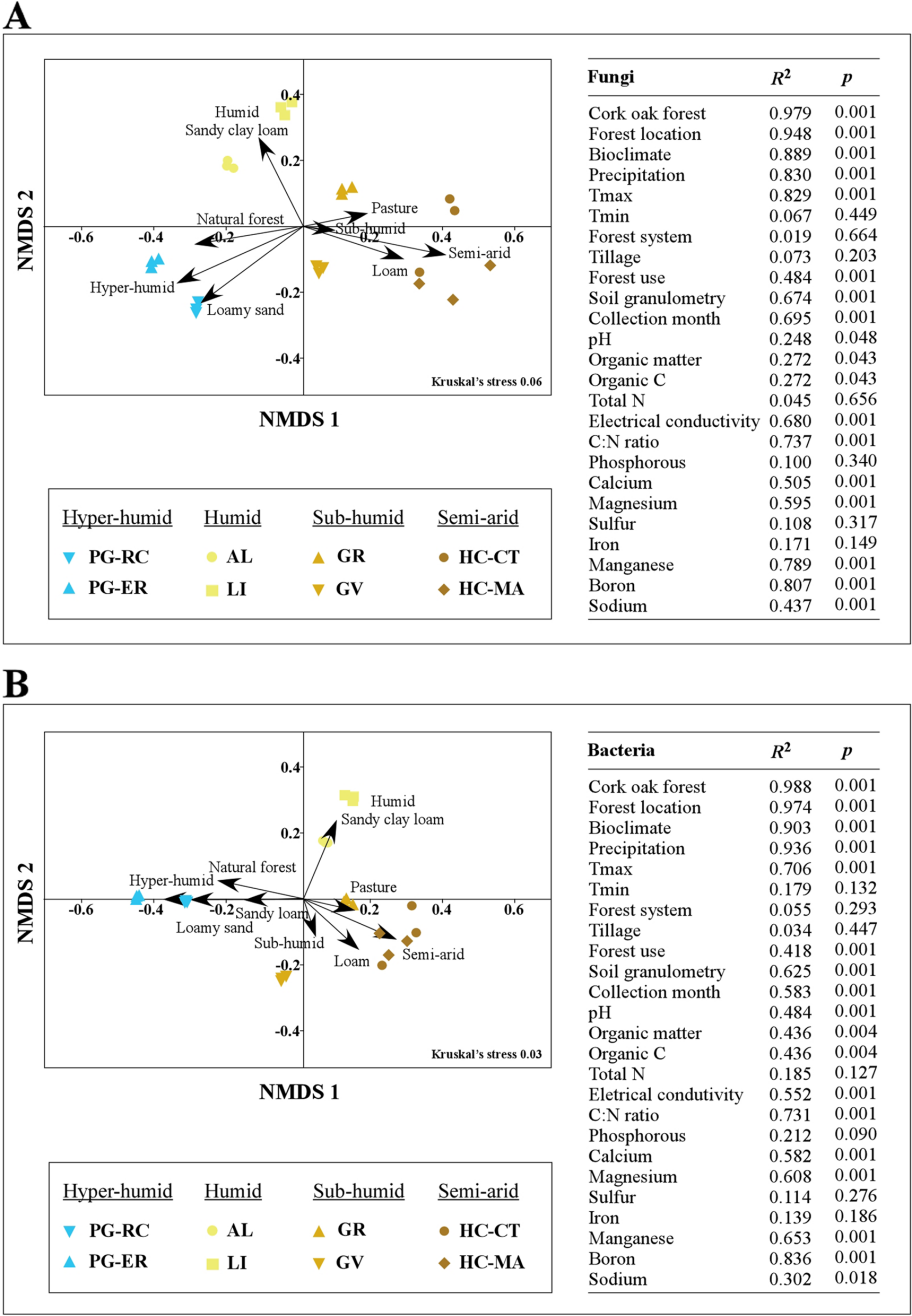
Evaluation of the main environmental factors affecting fungal (**A**) and bacterial (**B**) diversity. Non-metric multidimensional scaling (NMDS) analyses were performed using Bray-Curtis dissimilarity coefficients. Each symbol represents a different soil sample (three samples from each forest), with colours representing different forest bioclimates. Environmental variables were fitted onto the NMDS ordination. The arrows directions indicate positive correlations between continuous environmental factors and microbial assemblages. Tmax represents the maximum temperature in the warmest month, Tmin is the minimum temperature in the coldest month), Organic C is the organic carbon, Total N is the total nitrogen and C:N ratio is the carbon:nitrogen ratio. Only those factors that were significantly correlated with NMDS ordination axes (*p* < 0.05) are represented

The influence of different edaphoclimatic factors (Table S1 and S2) in shaping fungal and bacterial communities was evaluated by fitting the environmental factors onto a NMDS ordination plot (Fig. 3). NMDS analyses shown a good representation of the distribution of microbial communities (Kruskal’s stress < 0.2), revealing that they grouped differently according to the sampled forest, location and bioclimate. The microbial communities from semi-arid regions were the most distant from the other communities in moister regions. These results were corroborated by ANOSIM analyses that revealed highly dissimilar communities in different bioclimate groups (fungi: *R* = 0.954, *p* = 0.001; bacteria: *R* = 0.935, *p* = 0.001). These results were reinforced by homogeneity of group dispersions (fungi: F-value = 44.911; *p* = 4.52e^− 09^; bacteria: F-value = 12.281; *p* = 8.87e^− 05^). Less dissimilarities were found among humid and sub-humid forests (fungi: *R* = 0.783, *p* = 0.001; bacteria: *R* = 0.780, *p* = 0.001), as well as between sub-humid and semi-arid bioclimates (bacteria: *R* = 0.665, *p* = 0.001). Communities between all the other bioclimates were almost completely dissimilar *(R* ≥ 0.941, *p* = 0.001 for both microbial communities). These results suggest that particular drivers could have specifically shaped the microbial communities in arid and humid environments.

**Fig. 4:**
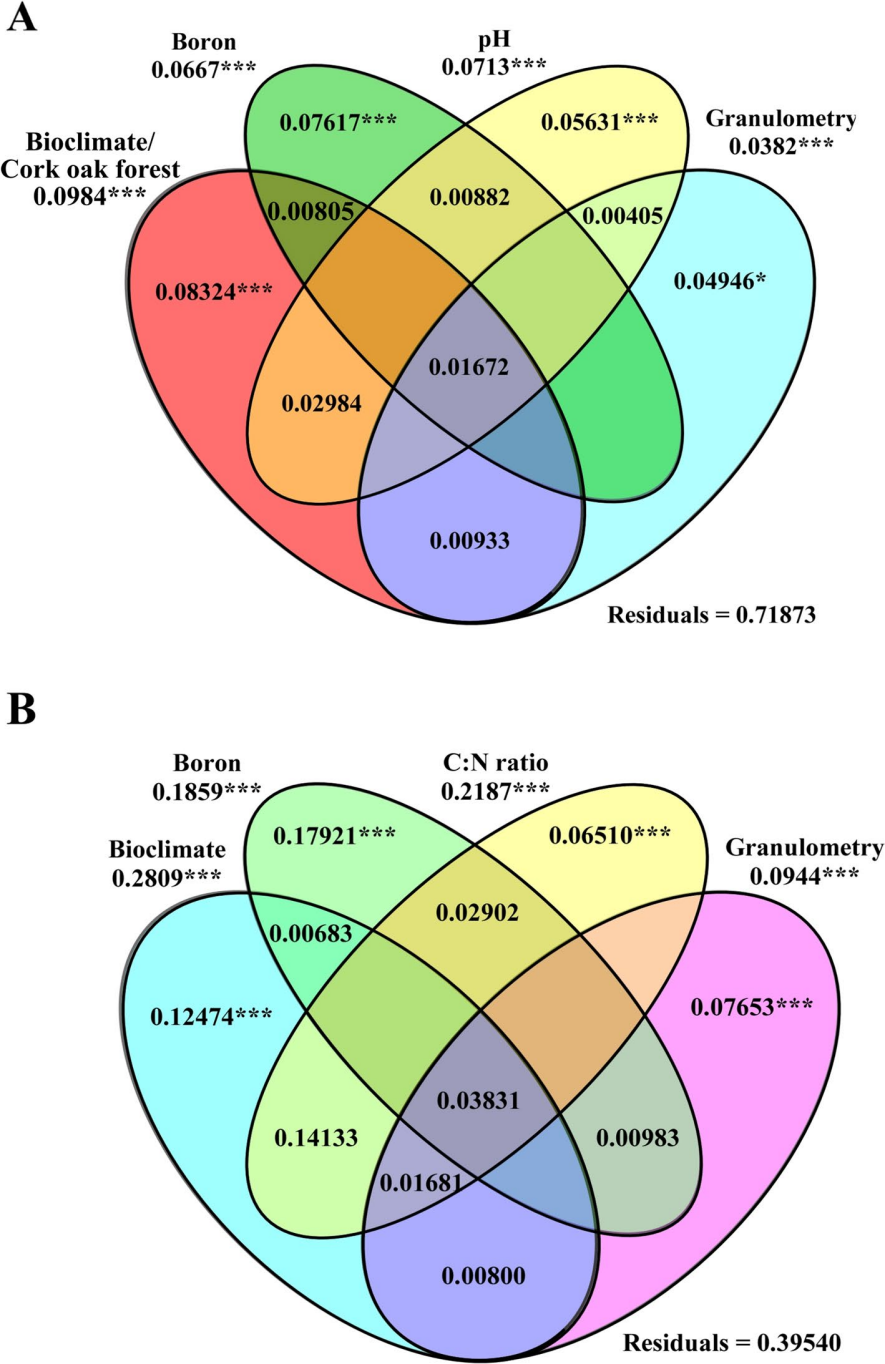
Contribution of edaphoclimatic factors in shaping fungal (**A**) and bacterial (**B**) communities. The most parsimonious model resulting from redundancy analysis is shown. The best explanatory variables were identified and their combined contribution for microbial shaping is displayed. Statistically significance values based on 999 permutations are represented as *** *p* < 0.001, ** *p* < 0.01 and *p < 0.05

According to the environmental factors fitted to NMDS ordination plot, most of the tested edaphoclimatic parameters influenced the microbial communities, with exception of ‘Tmin’, ‘forest system’, ‘tillage’, and ‘total N, P, S, Fe’ (Fig. 3). For both microbial communities, the highest *R*^2^ values were found for ‘cork oak forest’, ‘forest location’, ‘bioclimate’, and ‘precipitation’ factors (all above *R*^2^ > 0.8), but ‘Tmax’, ‘boron’ and ‘C:N ratio’ (in both communities) and ‘manganese’ (in fungal communities) were also particularly relevant (*R*^2^ > 0.7). The importance of bioclimate in shaping fungal and bacterial communities is in line with previous studies that revealed the influence of climate factors in bacteria [75] and ectomycorrhizal tips distribution in cork oak soils [28]. In the present work, the redundancy analysis (RDA) also revealed ‘bioclimate’ as the factor that better explained the differences among microbial communities (Fig. 4). A higher contribution of ‘bioclimate’ was found when considering the variation of bacterial communities (*R*^2^ = 0.903, *p* = 0.001, NMDS; 28.1% of variation, *p* = 0.001, RDA) in relation to variation of fungal communities (*R*^2^ = 0.889, *p* = 0.001, NMDS; 9.8% of variation, *p* = 0.001, RDA). However, ‘bioclimate’ and ‘cork oak forest’ were collinear variables (variables are correlated) for fungal community and these results should be taken with caution. Detected differences could be related with the different role of drier environments in shaping bacterial and fungal communities. While driest samples presented a significant reduction on fungal richness and diversity, bacterial communities increased both ecological parameters in semi-arid forests (Fig. 1). These results agree with other studies that revealed a much stronger impact of drought on bacterial than on fungal networks [89].

Besides ‘bioclimate’, both microbial communities were strongly affected by ‘cork oak forest’ and distance among cork oak forests (‘forest location’), both at *R*^2^ > 0.947, *p* = 0.001 (NMDS). But, neither of these factors revealed to significantly contribute to the shaping of bacterial community (Fig. 4). For shaping fungal communities besides collinear variables ‘bioclimate’ or ‘cork oak forest’, RDA displayed soil ‘pH’, ‘boron’ and ‘granulometry’ as playing a significant role in fungal community shaping (7.1%, *p* = 0.001, 6.7 and 3.8%, *p* = 0.001, respectively). All the other environmental factors were found to not contribute significantly for shaping fungal communities. A different picture was revealed by the factors that mostly shape bacterial communities (Fig. 4). The ‘C:N ratio’ and ‘boron’ factors played a major role in shaping bacterial communities (21.9%, *p* = 0.001 and 18.6%, *p* = 0.001, respectively), followed by soil granulometry (9.4%, *p* = 0.001). Altogether, ‘bioclimate’ and these three factors explained more than 60% of bacterial variation. This contrasts with the significant factors that shape fungal communities (‘bioclimate/cork oak forest’, ‘pH’, ‘boron’ and ‘granulometry’) that together only explained 27.4% of fungal variation. These results suggest that bacterial and fungal communities are shaped by different edaphoclimatic features at distinct levels, as previously reported by Shen et al. [90]. These authors suggested that pH and temperature were the best predictors for bacterial and fungal composition, respectively. In the present work, communities were not significantly affected by temperature alone, but bioclimate was distinguished as the main driver of bacterial communities. Interestingly, soil pH only explained variations detected on fungal community and was not significant for bacterial composition. This contrasts with multiple studies that report pH as the most important driver for bacterial diversity (e.g. [90, 91]). Detected differences could be related with the influence of other soil factors for shaping bacterial community. On the other hand, pH was found to be a significant factor influencing arbuscular communities [92] and was considered as the second most important driver of fungal communities on cork oak forests in Morocco, as reported by Maghnia et al. [29]. In the present work, pH was also the second most relevant driver for structuring studied fungal communities. Other edaphic features were also relevant for driving microbial communities, like it was the case of granulometry (for both communities). Accordingly, distinct soil granulometry contributed to different fungal communities in cork oak forests [26] and impacted bacterial diversity [93]. In addition, the structure of microbial communities was conditioned by ‘boron’ (both communities) and ‘C:N ratio’ (bacterial community). Boron is considered as a micronutrient essential for plant growth, but in high doses is known to influence microbial community composition by inhibiting soil microbial respiration [94]. However, fungi have been reported as more sensitive to boron in relation to bacteria [95], which contrasts with the finding of the strongest influence of boron levels on driving bacterial assembling when compared to its effect on fungal community shaping. Also, C:N ratio (representing the soil nutrient availability) modulates the bacterial community, as detected in different soil systems (e.g. [96, 97]). Although in the present work different land management practices (forest use and tillage) revealed none or little direct impact on soil microbial communities, they may have an indirect influence due to their influence on different soil properties, such as pH, carbon, nitrogen and phosphorus [98, 99]. The prominent influence of ‘bioclimate’ for the structure of microbial communities dictates the urgent need to understand the impact of climate change on microbial communities and final outcomes for cork oak forests sustainability.

### Correlation of microbial communities in different bioclimates

**Fig. 5:**
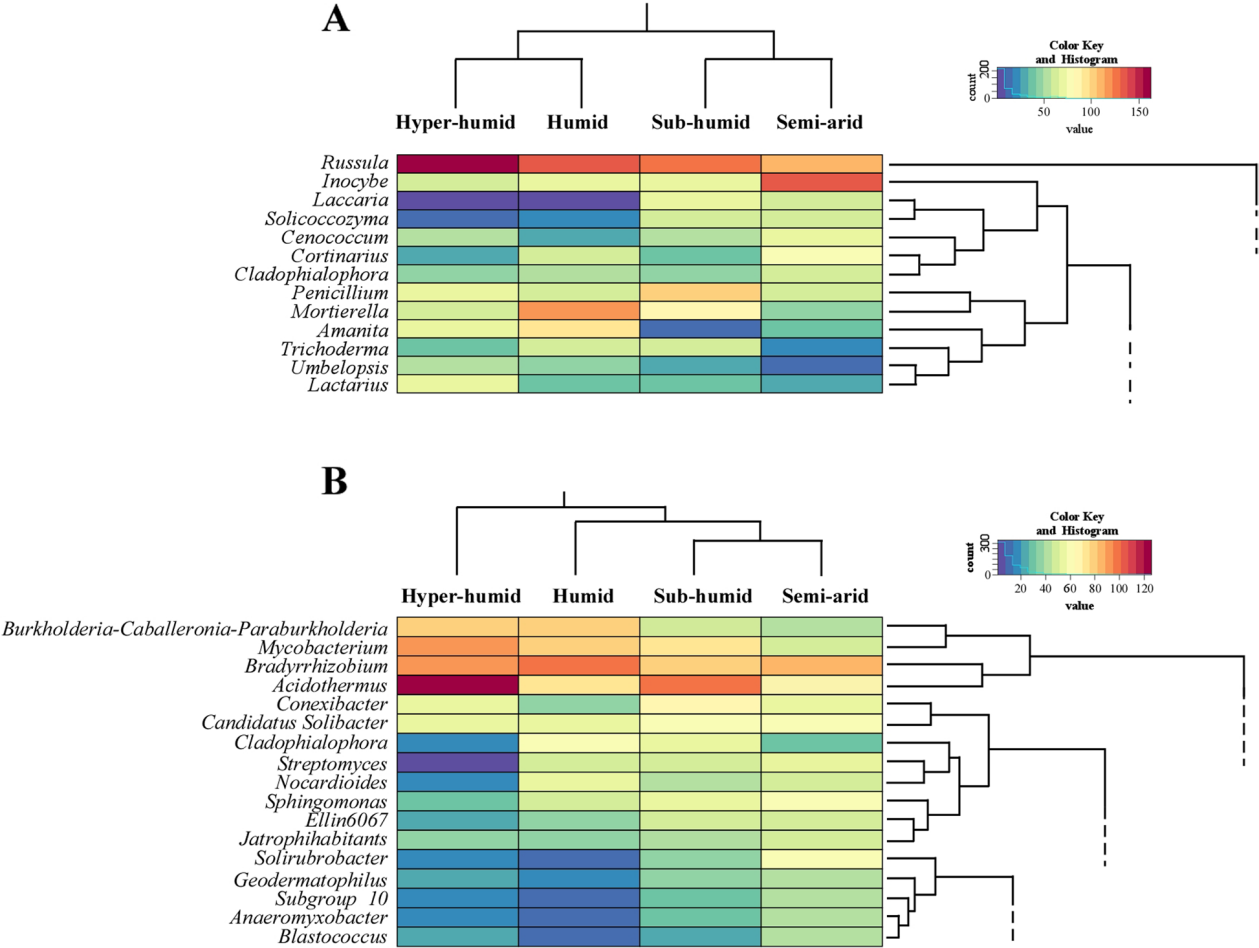
Heatmap depicting the most abundant core fungal (**A**), and bacterial (**B**) genera present in each bioclimate. Core communities were assessed considering those ASVs present in all bioclimates and classified up to genus level. Color represents differences in abundance, where dark red represents high abundance and dark blue low abundance. This figure is a detail of the heatmap depicting all core genera represented Fig. S7

The microbial taxa present in all soil samples included 102 fungal genera and 188 bacterial genera (core communities), which presented different abundances in distinct forest bioclimates (Fig. S7). Among fungi, many core ECM genera (*Russula*, *Inocybe*, *Laccaria*, *Cenococcum*, *Amanita*, *Lactarius*, *Tuber*, *Sebacina*, *Tomentella* and *Hebeloma*) have been previously described as core genera of Mediterranean cork oak forests [82]. The abundance of genera from the core fungal community in hyper-humid and humid forests clustered together, differently from the cluster formed by genera abundance in sub-humid and semi-arid forests. In contrast, the abundance of core bacterial community found in sub-humid and semi-arid bioclimates were the most similar, different from those present in humid forests, and even more dissimilar from the ones present in hyper-humid forests. The most abundant core genera were *Russula* (Russulaceae, 22% of core reads), *Inocybe* (Inocybaceae, 10%), *Mortierella* (Mortierellaceae, 8%), *Penicillium* (Aspergillaceae, 7%) and *Amanita* (Amanitaceae, 5%) (Fig. 5A). Following the general trend (Fig. 2A), *Russula*, *Mortierella* and *Amanita* decreased their presence from most humid to most arid bioclimates, which was particularly evident in *Russula* genus. In contrast, *Inocybe* and *Penicillium* were more present in semi-arid and sub-humid forests, respectively. Regarding the core bacterial community, the most abundant core genera were *Acidothermus* (*Acidothermaceae*, 11% of core reads), *Bradyrhizobium* (*Xanthobacteraceae*, 10%), *Mycobacterium* (*Mycobacteriaceae*, 7%) and *Burkholderia-Caballeronia-Paraburkholderia* (*Burkholderiaceae*, 5%) (Fig. 5B). These genera were mostly correlated with the most humid bioclimates. The families of all these bacterial genera were already reported as core families in cork oak forests [75].

**Fig. 6:**
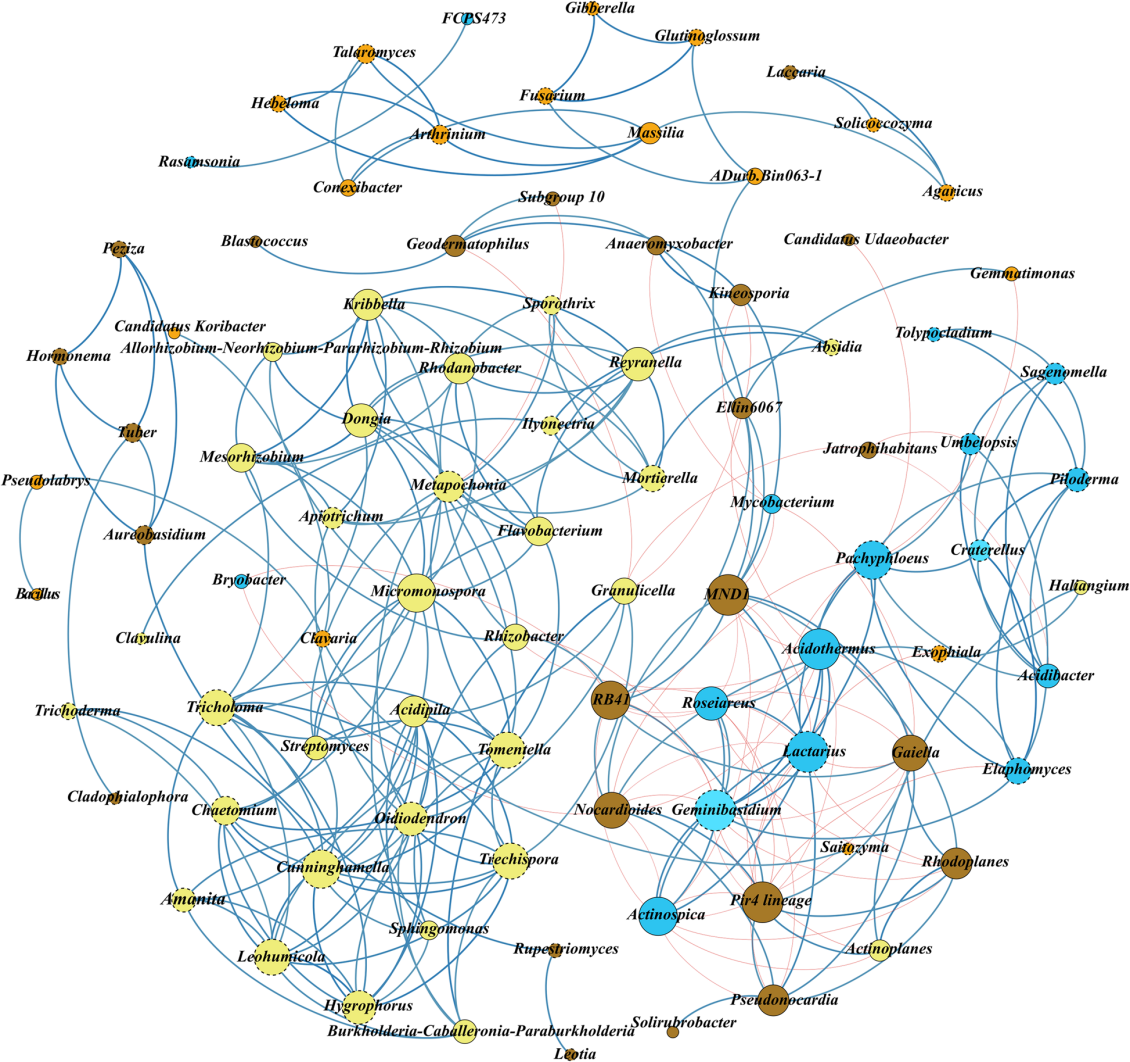
Co-occurrence network of fungal and bacterial biomarkers of different bioclimates. Pearson’s correlation coefficient was calculated for the top 50 genus displayed in LEfSe (R^2^ = 0.75, *p* < 0.05). Fungal nodes are represented by dashed circles and bacterial nodes by full circles. Size of the node corresponds to node degree (bigger nodes represent higher number of connections). Nodes are colored to represent biomarkers from different bioclimates (Hyper-humid in blue, Humid in yellow, Sub-humid in orange and Semi-arid in brown). Weighted edges in blue represent positive correlations and red represents negative correlations. Details of Pearson correlation matrix can be found in Fig. S9

For identifying biomarker taxa for each bioclimate, a linear discriminant analysis was performed with all dataset. Different fungal and bacterial genera revealed to be biomarkers for the analysed bioclimates at a LDA score higher than 4 (Fig. S8). A total of 49 fungal (10 hyper-humid; 18 humid; 11 sub-humid; 10 semi-arid; Fig. S8A) and 69 bacterial genera (10 hyper-humid; 23 humid; 10 sub-humid; 26 semi-arid: Fig. S8B) were defined as biomarkers with statistical significance (*p* < 0.05). Most of them (34 fungal and 66 bacterial taxa) were also identified as core genera, reinforcing the previous suggestion that specific bioclimates may favour the colonization by certain genera, even when they are generally widespread in all cork oak soils. As expected, correlations between fungal and bacterial biomarkers from a specific bioclimate were positively correlated, mainly concerning those from the most extreme (hyper-humid and semi-arid) bioclimates (Fig. 6; Fig. S9). Also, fungal biomarkers from the hyper-humid forests revealed the most negative correlations with bacterial biomarkers from other forests, particularly from semi-arid forests. Although not so evident the same trend was observed for bacterial in relation to fungal biomarkers. This result suggests that genera found in most humid (hyper-humid and humid) bioclimates are not prone to develop in the most arid (sub-humid and semi-arid) ones and vice versa. Interestingly, certain biomarker fungi revealed a complementary correlation with bacterial biomarkers from all the other bioclimate. For example, *Clavulina* (humid biomarker) was positively correlated with all biomarker bacteria from all bioclimates, except from those of hyper-humid. On the other hand, *Lactifluus* (humid biomarker) displayed positive correlation with most humid bacterial biomarkers and negative from those of drier forests. The same was found for certain biomarker bacteria (e.g.*, Burkholderia-Caballeronia-Paraburkholderia* vs. *Sphingomonas*). These results suggest that bioclimate modulates a complex co-occurrence network within both communities, determining microbial interactions with possible outcomes to forest sustainability. The recognition of physical and molecular fungal-bacterial interactions for plant health and for ecosystem functioning has been reported [13, 14]. For example, fungal communities were recently described to have an effect on bacterial composition in deadwood [100].

**Fig. 7:**
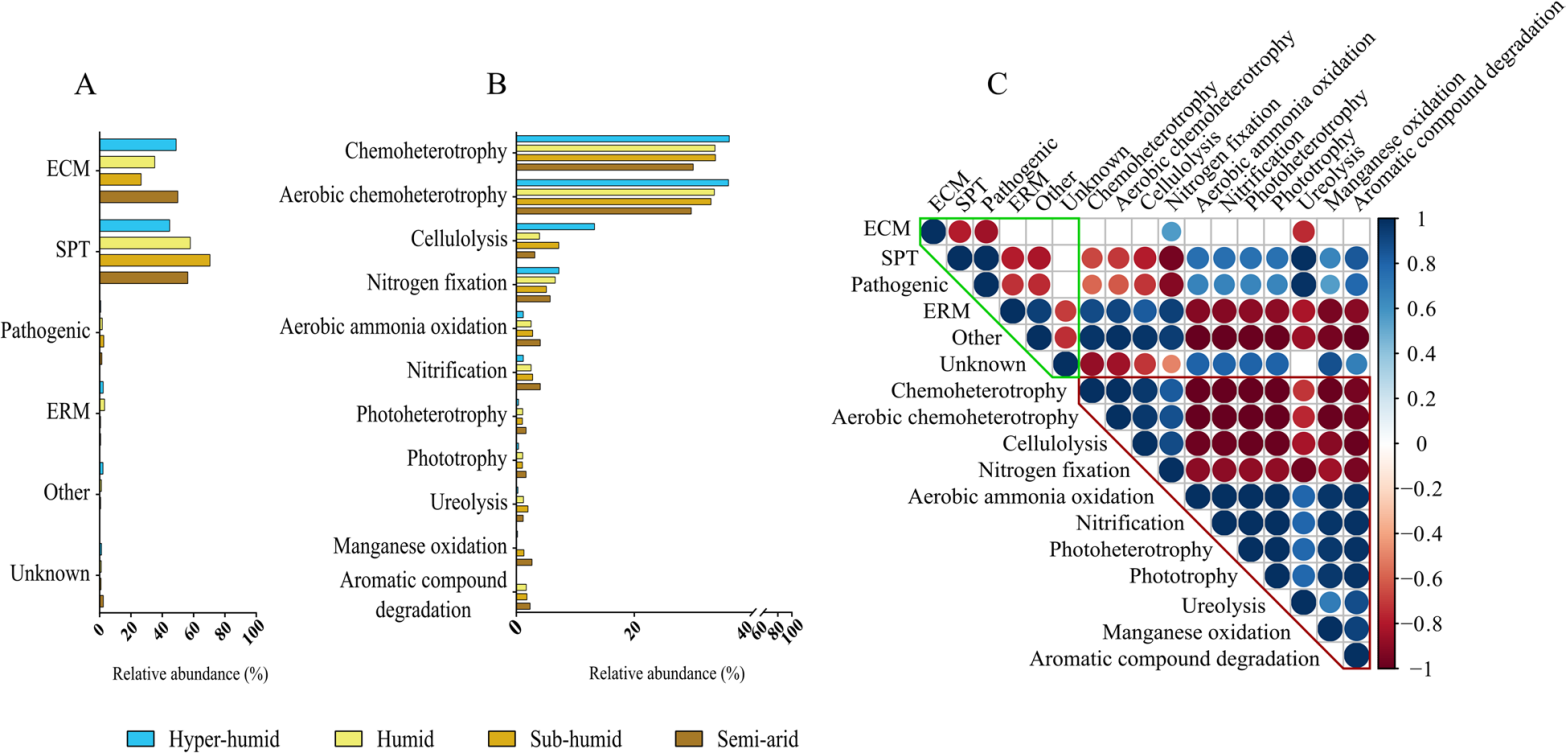
Relative abundance of functional groups of fungal (**A**) and bacterial (**B**) biomarkers in the different bioclimates and Pearson’s correlation matrix between functional groups (**C**). Only fungal and bacterial biomarkers obtained by LEfSe were used. Only more abundant bacterial functional groups are displayed in B. Full list of bacterial functional groups can be found in Table S5. ECM refers to ectomycorrhizae, SPT to saprotrophs and ERM to ericoid mycorrhiza. Blue and red circles represent statistically significant positive and negative correlations at *p* < 0.05, respectively. Color intensity is proportional to the correlation coefficients and circle size to statistical significance

The ecological functions of identified fungal and bacterial biomarkers in the ecosystem were predicted using *FunGuild* and *FAPROTAX*, respectively, and the relative abundance in each bioclimate is displayed (Fig. 7). The main ecological functions of biomarker fungi were as ectomycorrhizal (ECM) and saprophytic (SPT) fungi, being ECM prevalent in the most extreme environments (hyper-humid and semi-arid bioclimate, Fig. 7A). Indeed, ECM distribution have been related with climatic conditions (e.g., [101]), including in cork oak forests [27, 28]. The identified bacterial biomarkers were mainly related with chemoheterotrophy (33.30%) and aerobic chemoheterotrophy (32.97%). Bacteria with these ecological functions presented significantly higher abundance in hyper-humid than in semi-arid bioclimates (Fig. 7B; Table S5). Also, bacteria that perform cellulolysis represented 6.63% of total abundance and presented significantly higher abundance in hyper-humid bioclimate compared with all other bioclimates. These results suggest that different functional bacterial groups are thriving in different bioclimates, being the hyper-humid bioclimates more related with plant matter decomposition. When correlating different ecological functions within microbial communities (Fig. 7C), a significant negative correlation was observed among ECM and SPT fungi. Besides, these fungal guilds correlated differently with all the other bacterial functions. The different lifestyle of ECM and SPT and distinct nutrient acquisition strategies are able to create soil niches with discrete nutrient provisions that allow the propagation of bacteria with distinctive metabolic features [102]. Other ECM have been highly correlated with well-known mycorrhizae helper bacteria, and also with some not so well described bacteria, which reinforces the importance of fungi-bacteria interactions for forest sustainability [16, 103]. These results suggest once more the complex and delicate balance that occurs among microbial communities that is strongly dependent on bioclimate.

## Conclusions

Soil microbiomes are essential to maintain forest health and sustainability. Their role will be even more important under the pressure of ongoing climate changes. Among others (pH, boron levels, granulometry for fungi; C:N ratio, boron levels, granulometry for bacteria), bioclimate was the factor that contributed the most to shaping bacterial communities, while bioclimate/cork oak forest influences the structure of fungal communities in cork oak forests. These reinforces the concerns about these forests’ sustainability under a climate changing scenario. Forest soils with less water availability can develop richer and more abundant bacterial communities in comparison to fungal communities. However, differences on specific taxa abundance among bioclimates are more notable in fungi than in bacteria, raising the question about the effects of bioclimate in both microbial communities. The higher contribution of bioclimate for shaping bacterial communities predicts a higher impact of climate change on these compared to fungal communities. Final outcomes to cork oak forest sustainability will be dictated by the complex microbial network occurring between fungi and bacteria. More studies on specific microbial interactions will provide valuable information about which can be further explored for the protection of cork oak forests.

## Supplementary Information

**Additional file 1.**

**Additional file 2.**

## Data Availability

Raw reads for each sample from both microbial communities were deposited in NCBI Sequence Read Archive (http://www.ncbi.nlm.nih.gov/sra) under BioProject number PRJNA744048.
