# Supplementary material for: The influence of bioclimate on soil microbial communities of cork oak
Source: BMC Microbiol. 2022 Jun 23;22:163. doi: 10.1186/s12866-022-02574-2 (PMC9219136; doi:10.1186/s12866-022-02574-2)
Supplement: Supplementary file 1 — Additional file 1. [file 12866_2022_2574_MOESM1_ESM.docx]

**Table S1**. Cork oak forests used for soil sampling. Bioclimate was accessed by Emberger index and climatogram (Emberger, 1932, 1955; Haghighi et al., 2020), as described in *Material and methods* section. P corresponds to mean total precipitation, Tmax corresponds to the mean maximum temperature of the hottest month, and Tmin to the mean minimum temperature of the coldest month spanning 10 years (2006-2016) previous to sampling collection.

| Cork oak forest | Location | GPS coordinates | Collection date | Bioclimate | P | Tmax | Tmin | Forest system | Forest use | Tillage |
| --- | --- | --- | --- | --- | --- | --- | --- | --- | --- | --- |
| PG-ER | Parque da Peneda-Gerês  Parque da Peneda-Gerês | 41° 42’ N 8° 6’ W | May, 2017 | Hyper-humid | 1 391 | 26.72 | 3.29 | *Sobreiral* | Wild | Non-tilled |
| PG-RC |  | 41° 45’ N 8° 1’ W | July, 2017 | Hyper-humid | 1 378 | 26.61 | 2.80 | *Sobreiral* | Wild | Non-tilled |
| LI | Limãos | 41° 31’ N 6° 49’ W | April, 2017 | Humid | 995 | 29.44 | 2.29 | *Sobreiral* | Pasture | Tilled |
| AL | Alcobaça | 39° 27’ N 7° 55’ W | May, 2017 | Humid | 821 | 24.70 | 8.04 | *Sobreiral* | Wild | Non-tilled |
| GV | Gavião | 39° 27’ N 9° 2’ W | July, 2017 | Sub-humid | 844 | 30.45 | 6.65 | *Montado* | Pasture | Tilled |
| GR | Grândola | 38° 11’ N 8° 37’ W | May, 2017 | Sub-humid | 740 | 30.34 | 7.31 | *Montado* | Pasture | Tilled |
| HC-CT | Herdade da Contenda | 38° 2’ N 7° 0.5’ W | October, 2017 | Semi-arid | 632 | 32.47 | 4.86 | *Sobreiral* | Pasture | Non-tilled |
| HC-MA | Herdade da Contenda | 38° 2’ N 7° 1.9’ W | October, 2017 | Semi-arid | 641 | 32.45 | 4.68 | *Sobreiral* | Pasture | Non-tilled |


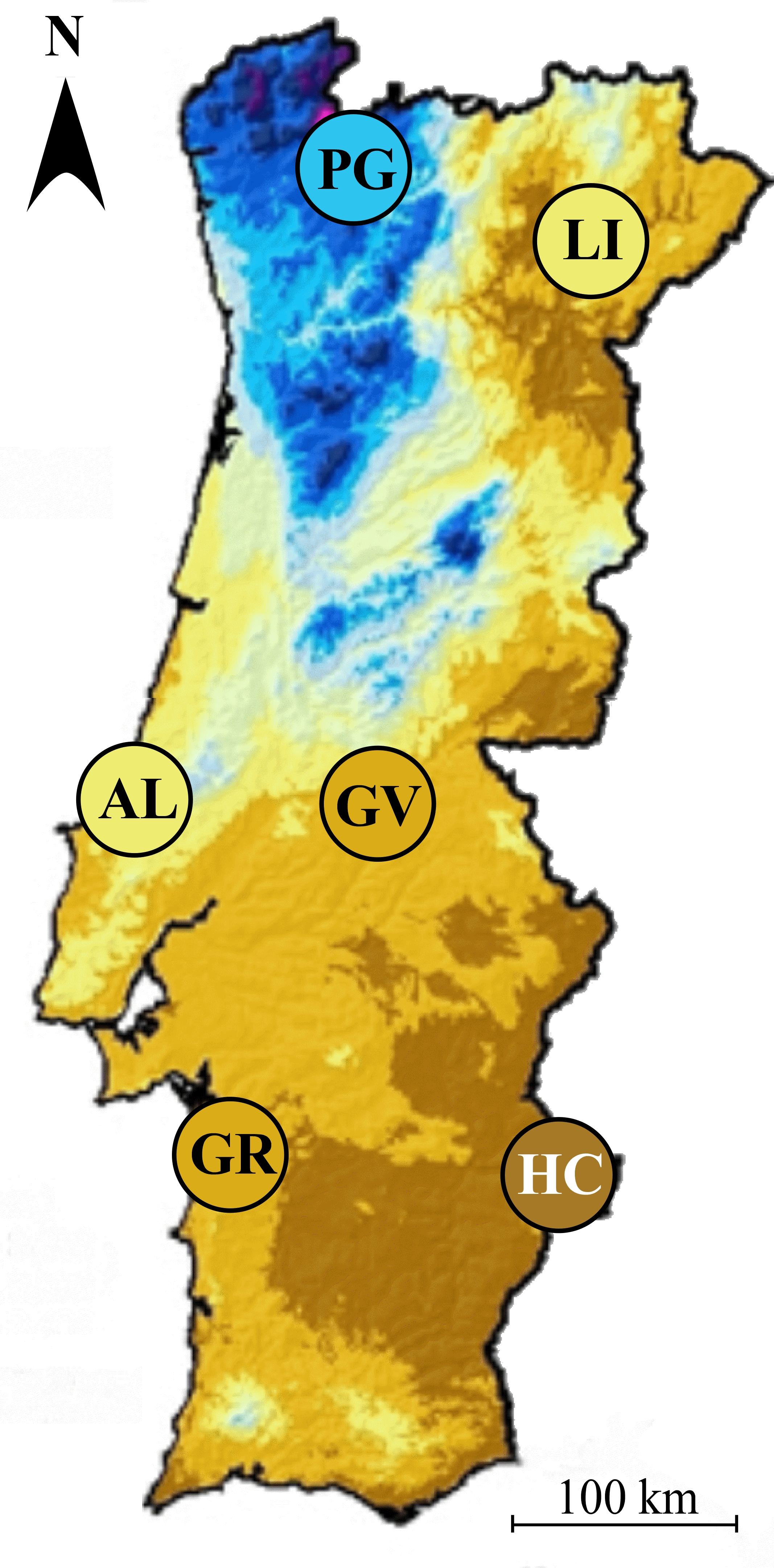


**Figure S1.** Cork oak forests used for soil sampling. Two cork oak samples were sampled from each bioclimate: hyper-humid (Parque Peneda Gerês, PG: two sampling sites PG-ER and PG-RC; in blue); humid (Limãos, LI; Alcobaça, AL; in yellow); sub-humid (Gavião, GV; Grândola, GR; in light brown) and semi-arid (Herdade Contenda, HC: two sampling sites HC-CT and HC-MA; in dark brown). Portugal annual accumulated precipitation chart was taken from https://www.ipma.pt/pt/educativa/tempo.clima.

**Table S2**. Physicochemical properties of soil samples collected from the eight studied Portuguese cork oak forests.

|  | Hyper-humid | |  | Humid | |  | Sub-humid | |  | Semi-arid | |
| --- | --- | --- | --- | --- | --- | --- | --- | --- | --- | --- | --- |
|  | **PG-ER** | **PG-RC** |  | **LI** | **AL** |  | **GV** | **GR** |  | **HC-CT** | **HC-MA** |
| Granulometry | Sandy  loam | Loamy sand |  | Sandy clay loam | Sandy clay loam |  | Loam | Sandy loam |  | Loam | Loam |
| pH (H_2_O) | 4.31 ± 0.01 | 5.18 ± 0.01 |  | 5.33 ± 0.01 | 6.15 ± 0.01 |  | 5.03 ± 0.01 | 5.16 ± 0.01 |  | 5.30 ± 0.01 | 5.49 ± 0.01 |
| pH (CaCl_2_) | 3.68 ± 0.1 | 3.96 ± 0.1 |  | 4.9 ± 0.1 | 5.90 ± 0.1 |  | 4.03 ± 0.1 | 4.59 ± 0.01 |  | 4.63 ± 0.1 | 4.76 ± 0.1 |
| Electrical conductivity  (μS/cm) | 176 ± 2 | 29 ± 2 |  | 607 ± 2 | 154 ± 2 |  | 141 ± 2 | 283 ± 2 |  | 118 ± 2 | 103 ± 2 |
| Organic matter (%) | 10.90 ± 0.01 | 11.59 ± 0.01 |  | 13.17 ± 0.01 | 3.53 ± 0.01 |  | 8.05 ± 0.01 | 5.01 ± 0.01 |  | 4.97 ± 0.01 | 5.86 ± 0.01 |
| Organic C (%) | 6.32 ± 0.04 | 6.72 ± 0.04 |  | 7.64 ± 0.04 | 2.05 ± 0.04 |  | 4.67 ± 0.04 | 2.91 ± 0.04 |  | 2.88 ± 0.04 | 3.40 ± 0.04 |
| Total N (%) | 0.29 ± 0.02 | 0.38 ± 0.02 |  | 0.52 ± 0.02 | 0.12 ± 0.02 |  | 0.23 ± 0.02 | 0.19 ± 0.02 |  | 0.25 ± 0.02 | 0.28 ± 0.02 |
| C:N ratio | 21.4 | 17.8 |  | 14.8 | 17.6 |  | 19.9 | 15.7 |  | 11.6 | 11.9 |
| Phosphorus (P_2_O_5_) (mg/kg) | 30.2 ± 0.4 | 15.0 ± 0.4 |  | 25.6 ± 0.4 | 6.8 ± 0.4 |  | 43.1 ± 0.4 | 28.9 ± 0.4 |  | 111.3 ± 0.4 | 48.0 ± 0.4 |
| Potassium (K_2_O) (mg/kg) | 86.2 ± 1.3 | 22.9 ± 1.2 |  | 123.0 ± 1.3 | 126.5 ± 1.2 |  | 292.1 ± 1.2 | 115.1 ± 1.3 |  | 163.5 ± 1.2 | 81.4 ± 1.3 |
| Calcium (CaO) (mg/kg) | 224.3 ± 0.9 | 130.5 ± 0.9 |  | 1221.4 ± 0.4 | 2403.9 ± 0.9 |  | 501.7 ± 0.9 | 1373.6 ± 0.9 |  | 917.7 ± 0.9 | 1056.7 ± 0.9 |
| Magnesium (MgO) (mg/kg) | 65.6 ± 0.4 | 34.6 ± 0.4 |  | 2794.8 ± 0.9 | 285.0 ± 0.4 |  | 168.0 ± 0.4 | 521.0 ± 0.4 |  | 336.2 ± 0.4 | 284.1 ± 0.4 |
| Sulfur (mg/kg) | 17.2 ± 0.1 | 4.5 ± 0.1 |  | 13.4 ± 0.1 | 7.1 ± 0.1 |  | 12.3 ± 0.1 | 7.4 ± 0.1 |  | 9.6 ± 0.1 | 7.2 ± 0.1 |
| Iron (mg/kg) | 209.0 ± 0.1 | 172.9 ± 0.1 |  | 350.4 ± 0.1 | 112.0 ± 0.1 |  | 234.4 ± 0.1 | 172.2 ± 0.1 |  | 162.9 ± 0.1 | 166.1 ± 0.1 |
| Manganese (MnAI) | 411.0 ± 0.0 | 260.5 ± 0.0 |  | 367.1 ± 0.0 | 327.8 ± 0.0 |  | 336.8 ± 0.0 | 651.2 ± 0.0 |  | 1057.9 ± 0.0 | 1084.9 ± 0.0 |
| Boron (mg/kg) | <0.03 | <0.04 |  | 0.41 ± 0.0 | 0.38 ± 0.0 |  | 0.09 ± 0.0 | 0.16 ± 0.0 |  | 0.11 ± 0.0 | 0.11 ± 0.0 |
| Sodium (mg/kg) | 12.5 ± 0.1 | 9.7 ± 0.1 |  | 22.8 ± 0.1 | 27.2 ± 0.1 |  | 19.5 ± 0.1 | 37.1 ± 0.1 |  | 15.7 ± 0.1 | 16.7 ± 0.1 |

**Table S3**. Processing of fungal and bacterial reads sequenced by *Illumina MiSeq*, using DNA obtained from cork oak soil samples as described in *Material and methods* section.

| Cork oak forest | Samples | Fungi | | | |  | Bacteria | | | |
| --- | --- | --- | --- | --- | --- | --- | --- | --- | --- | --- |
|  |  | **Raw read pairs** | **Merged reads** | **Reads clustered into ASV** | **Fungal sequences** |  | **Raw read pairs** | **Merged reads** | **Reads clustered into ASV** | **Bacterial sequences** |
|  |  |  |  |  |  |  |  |  |  |  |
| *PG-ER* | 1 | 101 991 | 87 560 | 84 494 | 83 579 |  | 58 968 | 50 302 | 31 235 | 31 197 |
|  | 2 | 131 390 | 114 161 | 110 686 | 109 119 |  | 58 745 | 50 575 | 28 306 | 28 291 |
|  | 3 | 136 239 | 118 193 | 114 895 | 113 676 |  | 61 173 | 52 598 | 30 343 | 30 318 |
|  | **Total** | **369 620** | **319 914** | **310 075** | **306 374** |  | **178 886** | **153 475** | **89 884** | **89 806** |
| *PG-RC* | 1 | 107 607 | 92 824 | 89 216 | 86 455 |  | 72 940 | 60 797 | 35 392 | 35 365 |
|  | 2 | 120 998 | 102 489 | 98 595 | 95 769 |  | 75 800 | 65 275 | 39 363 | 39 329 |
|  | 3 | 92 274 | 78 600 | 75 607 | 72 875 |  | 90 848 | 78 124 | 46 504 | 46 459 |
|  | **Total** | **320 879** | **273 913** | **263 418** | **255 099** |  | **239 588** | **204 196** | **121 259** | **121 153** |
| *LI* | 1 | 60 096 | 49 740 | 47 847 | 46 869 |  | 84 422 | 73 106 | 44 042 | 44 031 |
|  | 2 | 95 396 | 83 133 | 79 301 | 77 660 |  | 91 233 | 70 565 | 42 897 | 42 889 |
|  | 3 | 131 962 | 114 706 | 109 539 | 106 322 |  | 60 821 | 52 094 | 30 362 | 30 355 |
|  | **Total** | **287 454** | **247 579** | **236 687** | **230 851** |  | **236 476** | **195 765** | **117 301** | **117 275** |
| *AL* | 1 | 96 184 | 82 854 | 79 260 | 78 225 |  | 54 449 | 46 663 | 27 246 | 27 228 |
|  | 2 | 75 667 | 61 614 | 59 281 | 58 035 |  | 54 549 | 46 667 | 27 832 | 27 823 |
|  | 3 | 67 549 | 56 255 | 53 675 | 52 918 |  | 50 673 | 42 144 | 25 106 | 25 087 |
|  | **Total** | **239 400** | **200 723** | **192 216** | **189 178** |  | **159 671** | **135 474** | **80 184** | **80 138** |
| *GV* | 1 | 65 652 | 56 536 | 54 046 | 52 637 |  | 80 783 | 69 586 | 42 194 | 42 108 |
|  | 2 | 172 166 | 149 884 | 143 767 | 140 252 |  | 86 898 | 73 809 | 47 997 | 47 891 |
|  | 3 | 89 038 | 76 953 | 73 826 | 72 346 |  | 74 274 | 63 224 | 37 822 | 37 755 |
|  | **Total** | **326 856** | **283 373** | **271 639** | **265 235** |  | **241 955** | **206 619** | **128 013** | **127 754** |
| *GR* | 1 | 40 904 | 28 592 | 27 228 | 26 787 |  | 54 093 | 46 572 | 29 472 | 29 438 |
|  | 2 | 95 720 | 82 703 | 78 628 | 77 079 |  | 73 875 | 63 450 | 38 637 | 38 600 |
|  | 3 | 75 944 | 65 705 | 62 027 | 60 628 |  | 59 337 | 50 809 | 30 210 | 30 161 |
|  | **Total** | **212 568** | **177 000** | **167 883** | **164 494** |  | **187 305** | **160 831** | **98 319** | **98 199** |
| *HC-CT* | 1 | 118 817 | 103 422 | 101 022 | 83 828 |  | 61 520 | 52 585 | 29 757 | 29 718 |
|  | 2 | 73 055 | 61 423 | 59 277 | 57 302 |  | 58 840 | 46 420 | 27 740 | 27 712 |
|  | 3 | 115 853 | 102 293 | 100 018 | 97 746 |  | 59 417 | 51 160 | 31 049 | 31 026 |
|  | **Total** | **307 725** | **267 138** | **260 317** | **238 876** |  | **179 777** | **150 165** | **88 546** | **88 456** |
| *HC-MA* | 1 | 99 307 | 86 021 | 83 404 | 79 027 |  | 57 850 | 49 339 | 29 662 | 29 623 |
|  | 2 | 83 427 | 73 628 | 71 502 | 69 156 |  | 63 482 | 54 107 | 34 612 | 34 487 |
|  | 3 | 118 027 | 104 602 | 101 662 | 98 772 |  | 65 308 | 56 205 | 31 232 | 31 201 |
|  | **Total** | **300 761** | **264 251** | **256 568** | **246 955** |  | **186 640** | **159 651** | **95 506** | **95 311** |
| Total | | **2 365 263** | **2 033 891** | **1 958 803** | **1 897 062** |  | **1 610 298** | **1 366 176** | **819 012** | **818 092** |

**Table S4**. Detailed dataset of fungal (A) and bacterial (B) communities found in cork oak forest soils, obtained by sequencing *ITS2* or *V5-V7* (*16S*) barcodes, respectively. For avoiding bias introduced by different sequencing depths, total datasets were subsampled to samples with the least number of sequences (see details in the main text). The taxonomic distribution of all ASV is discriminated according to all identified taxa levels. See Excel file.


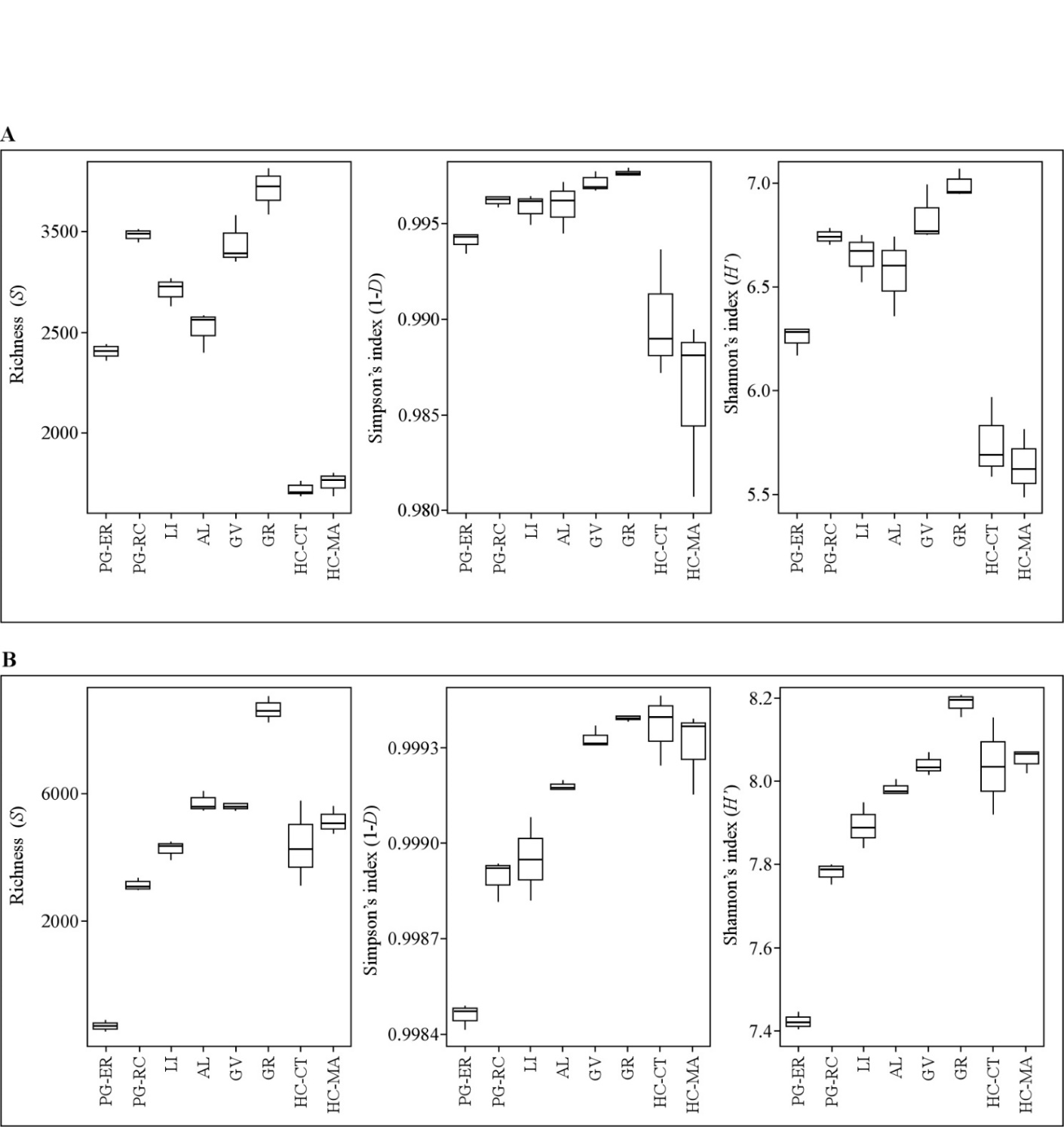


**Figure S2**. Diversity of fungal (A) and bacterial (B) communities found in each cork oak forest. *S* represents microbial richness, 1-*D* Gini-Simpson’s index and *H*’ Shannon’s index. Significant differences were not found (*p*> 0.05).


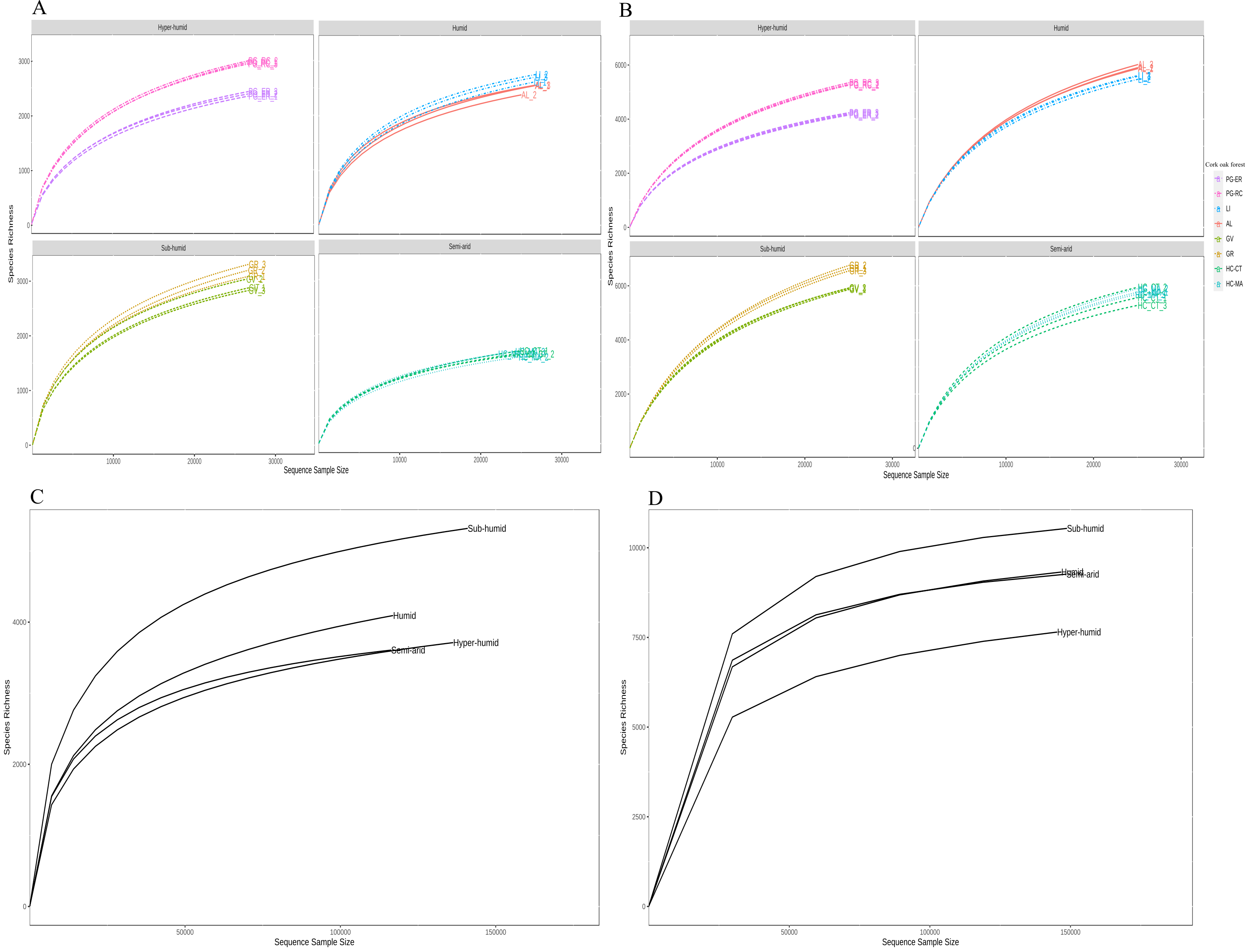


**Figure S3.** Rarefaction curves of fungal (A and C) and bacterial (B and D) subsampled datasets. Rarefaction curves per sample (A and B) and per bioclimate (C and D) are presented.


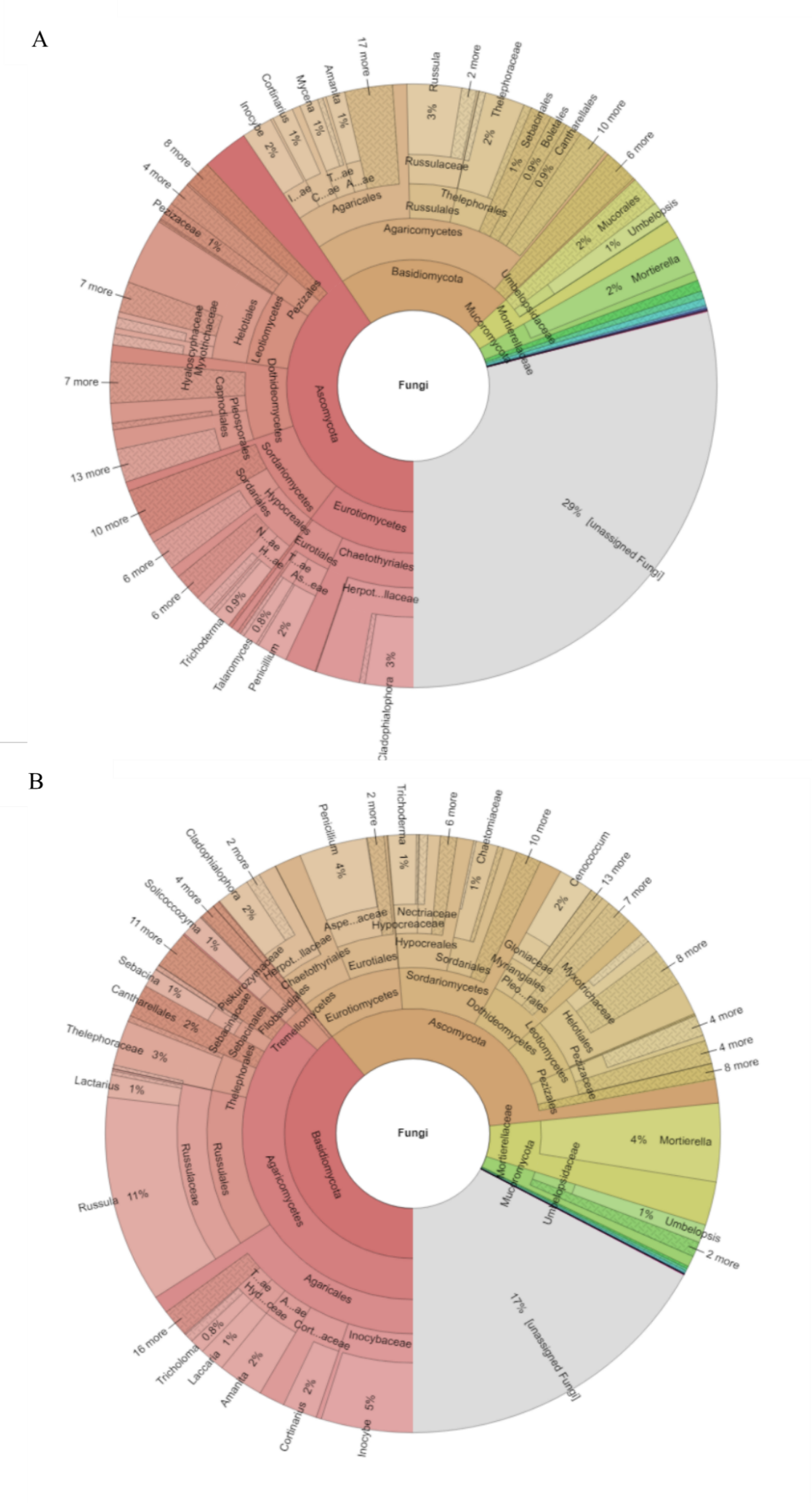
**Figure S4.** Krona charts of cork oak soil fungal community richness (A) and abundance (B).


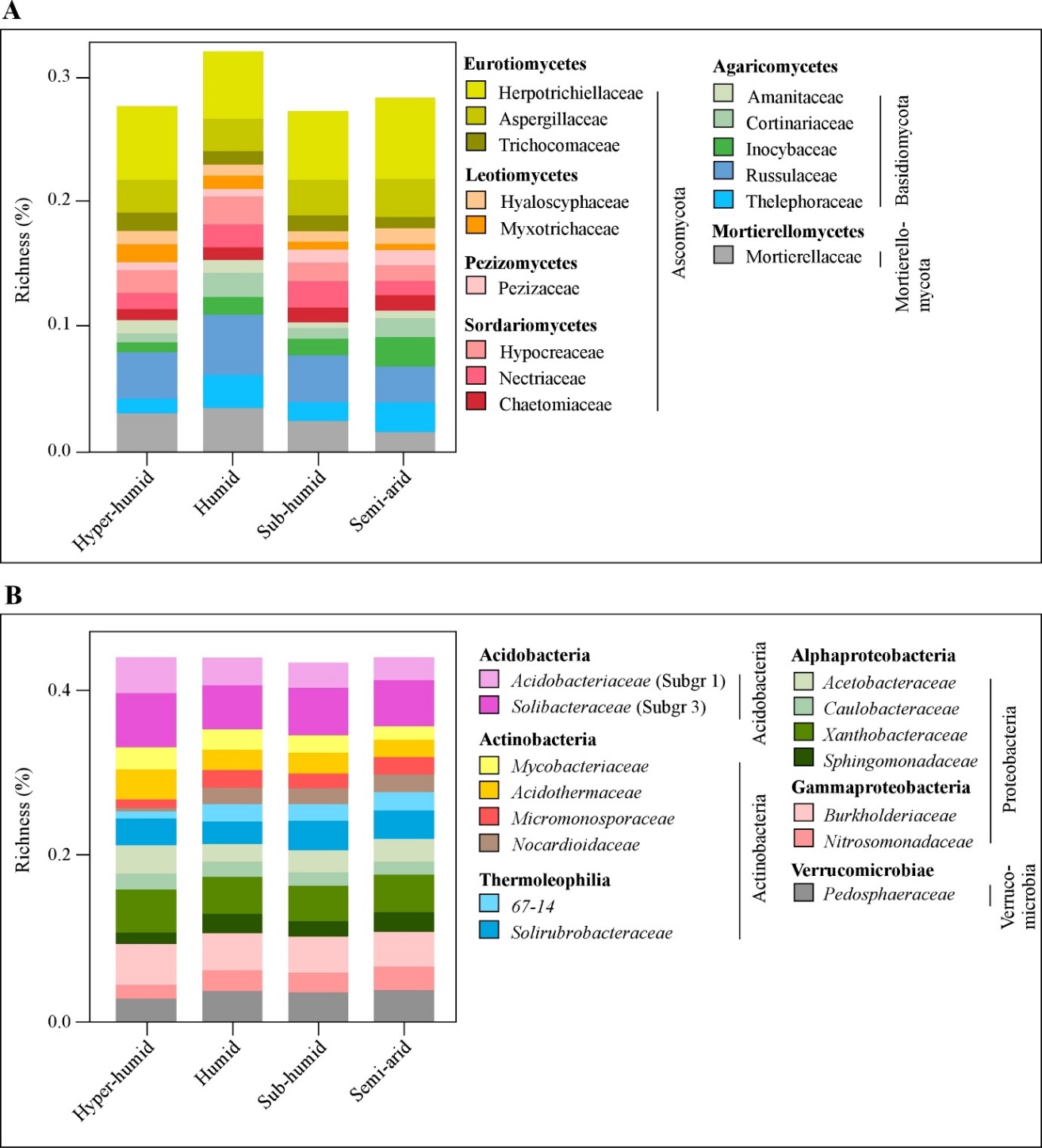


**Figure S5.** Mean relative richness of soil fungal (A) and bacterial (B) communities in forests from each bioclimate. Only those families that present a relative abundance higher than 1% (for fungi) and 2% (for bacteria) in every bioclimate are represented, being indicated the corresponding classes and phyla.


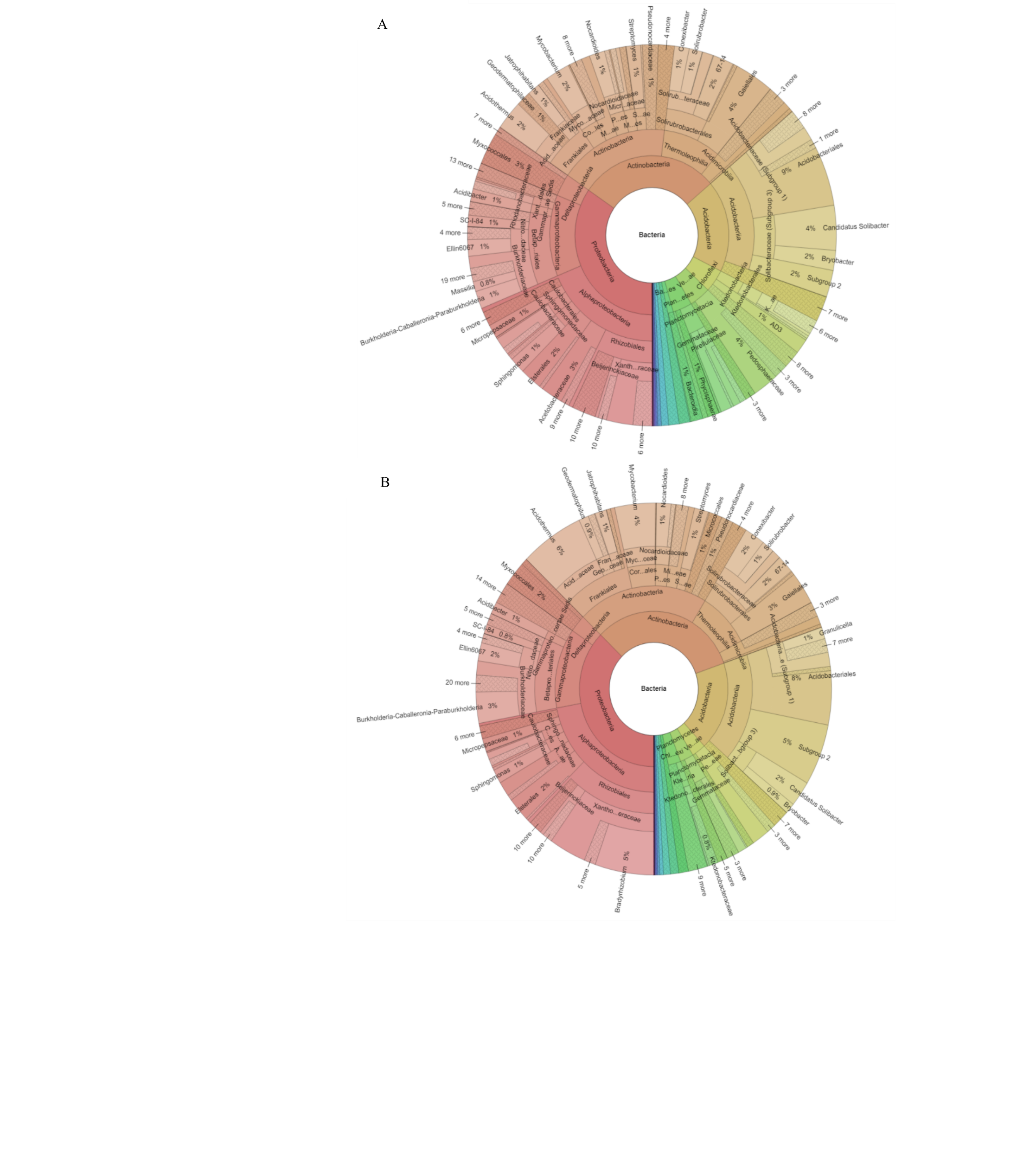


**Figure S6**. Krona charts of cork oak soil bacterial community richness (A) and abundance (B).

**
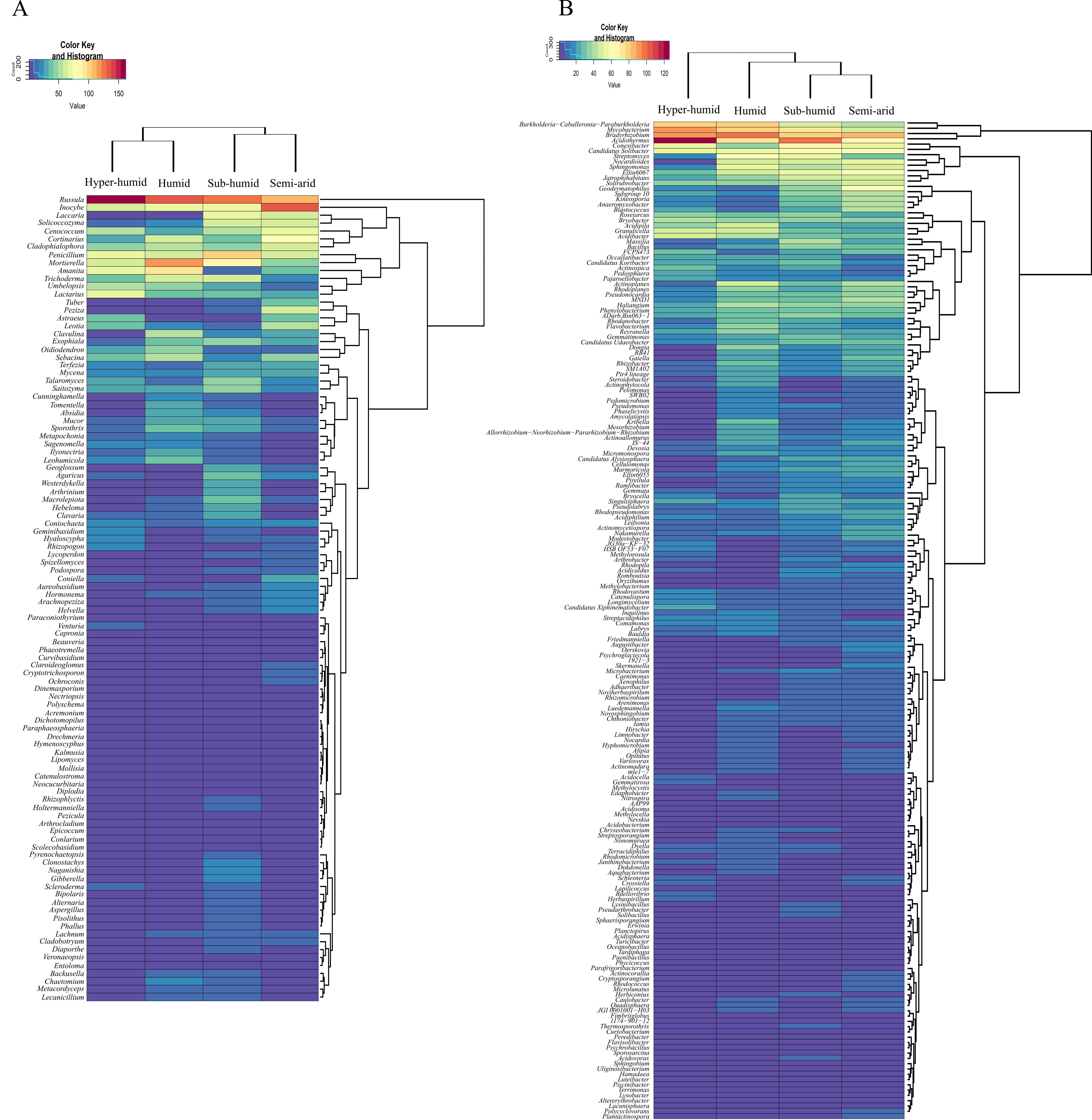
**

**Figure S7.** Heatmap depicting the abundance of core fungal (A) and bacterial (B) genera present in each bioclimate. Core communities were assessed considering those ASVs present in all bioclimates and classified up to genus level. Color represent differences in abundance, where dark red represents high abundance and dark blue low abundance. Details of the most abundant core genera are represented in the main text.

 
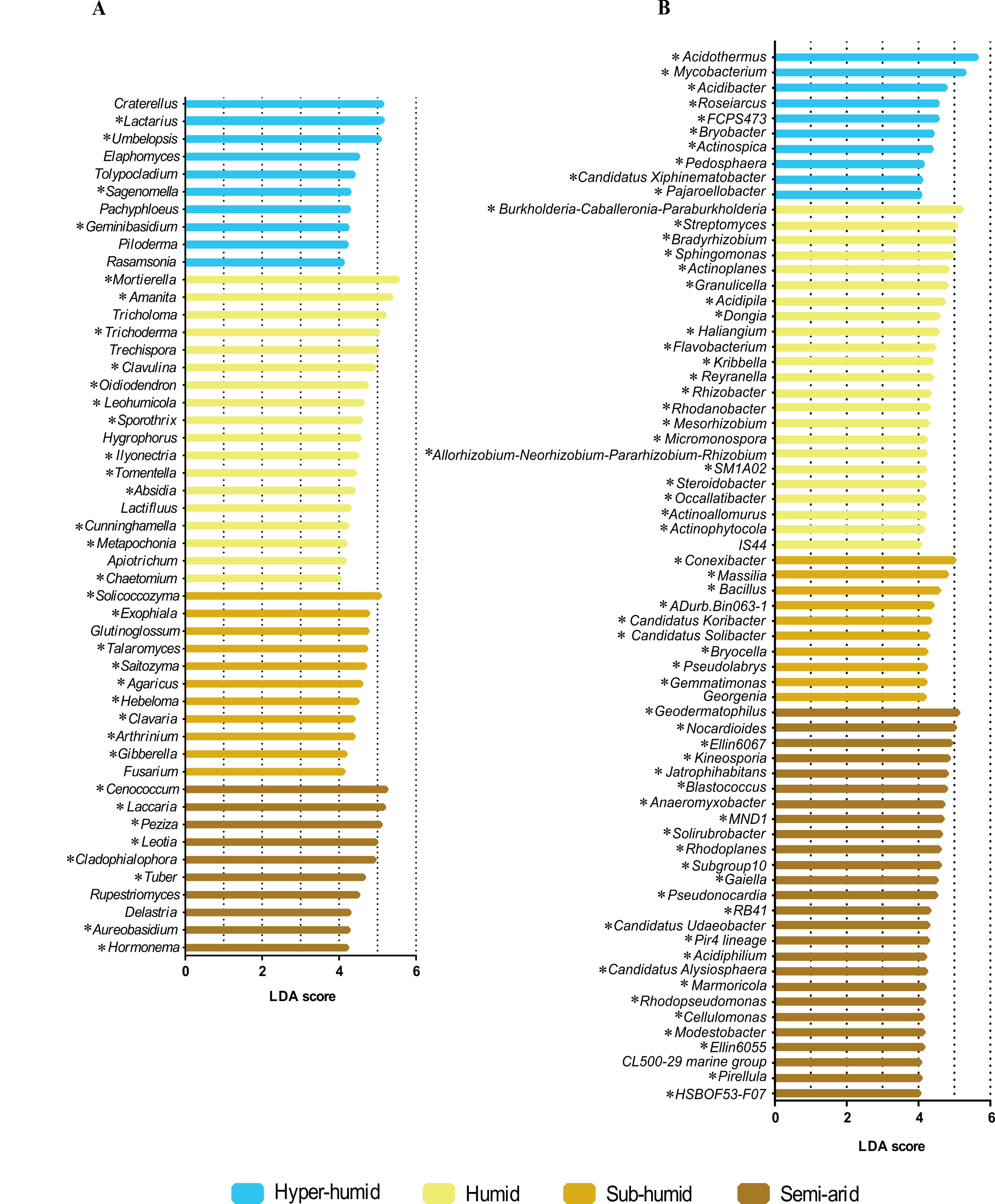


**Figure S8**. Linear discriminant analysis of fungal (A) and bacterial (B) genera among distinct bioclimates. Taxa were detected by Linear discriminant analysis Effect Size (LEfSe) analysis with a LDA threshold score of 4 (*p* < 0.05).

**Table S5.** Relative abundance (%) of functional groups of bacterial biomarkers in the different bioclimates. Functional classification was performed using FAPROTAX 1.2.4 and only bacterial biomarkers obtained by LefSe were used. Different letters correspond to statistically significant differences at *p*<0.05.

| Functional group | Hyper-humid | Humid | Sub-humid | Semi-arid |
| --- | --- | --- | --- | --- |
| Chemoheterotrophy | 36.104^a^ | 33.683^ab^ | 33.743^ab^ | 29.983^b^ |
| Aerobic chemoheterotrophy | 35.960^a^ | 33.563^ab^ | 32.987^ab^ | 29.654^b^ |
| Cellulolysis | 13.248^a^ | 3.928^b^ | 7.198^b^ | 3.12^b^ |
| Nitrogen fixation | 7.215 | 6.594 | 5.096 | 5.735 |
| Aerobic ammonia oxidation | 1.190 | 2.514 | 2.776 | 4.054 |
| Nitrification | 1.190 | 2.514 | 2.776 | 4.054 |
| Predatory or exoparasitic | 0.508 | 1.269 | 1.135 | 1.042 |
| Photoheterotrophy | 0.366 | 1.082 | 1.030 | 1.652 |
| Phototrophy | 0.366 | 1.082 | 1.030 | 1.652 |
| Animal parasites or symbionts | 0.346 | 0.239 | 0.192 | 0.119 |
| Ureolysis | 0.267 | 1.225 | 1.984 | 1.158 |
| Nitrate reduction | 0.249 | 0.968 | 0.549 | 1.130 |
| Nitrate respiration | 0.228 | 0.884 | 0.498 | 1.042 |
| Nitrogen respiration | 0.228 | 0.884 | 0.498 | 1.042 |
| Nitrate denitrification | 0.228 | 0.877 | 0.473 | 1.040 |
| Nitrite denitrification | 0.228 | 0.877 | 0.473 | 1.040 |
| Nitrous oxide denitrification | 0.228 | 0.877 | 0.473 | 1.040 |
| Denitrification | 0.228 | 0.877 | 0.473 | 1.040 |
| Nitrite respiration | 0.228 | 0.877 | 0.473 | 1.040 |
| Anoxygenic photoautotrophy S oxidizing | 0.228 | 0.877 | 0.473 | 1.040 |
| Anoxygenic photoautotrophy | 0.228 | 0.877 | 0.473 | 1.040 |
| Photoautotrophy | 0.228 | 0.877 | 0.473 | 1.040 |
| Manganese oxidation | 0.198 | 0.069 | 1.294 | 2.659 |
| Methylotrophy | 0.111 | 0.033 | 0.146 | 0.111 |
| Methanotrophy | 0.101 | 0.028 | 0.030 | 0.003 |
| Hydrocarbon degradation | 0.101 | 0.028 | 0.030 | 0.003 |

**Table S5.** *Continuation*

| Iron respiration | 0.081 | 0.089 | 0.257 | 0.162 |
| --- | --- | --- | --- | --- |
| Fermentation | 0.053 | 0.292 | 0.839 | 0.662 |
| Aromatic compound degradation | 0.026 | 1.697 | 1.802 | 2.337 |
| Human associated | 0.013 | 0.126 | 0.073 | 0.073 |
| Human pathogens all | 0.013 | 0.099 | 0.052 | 0.058 |
| Methanol oxidation | 0.010 | 0.004 | 0.116 | 0.108 |
| Chitinolysis | 0.002 | 0.024 | 0.017 | 0.024 |
| Human gut | 0.000 | 0.027 | 0.021 | 0.015 |
| Mammal gut | 0.000 | 0.027 | 0.021 | 0.015 |
| Xylanolysis | 0.000 | 0.007 | 0.023 | 0.005 |


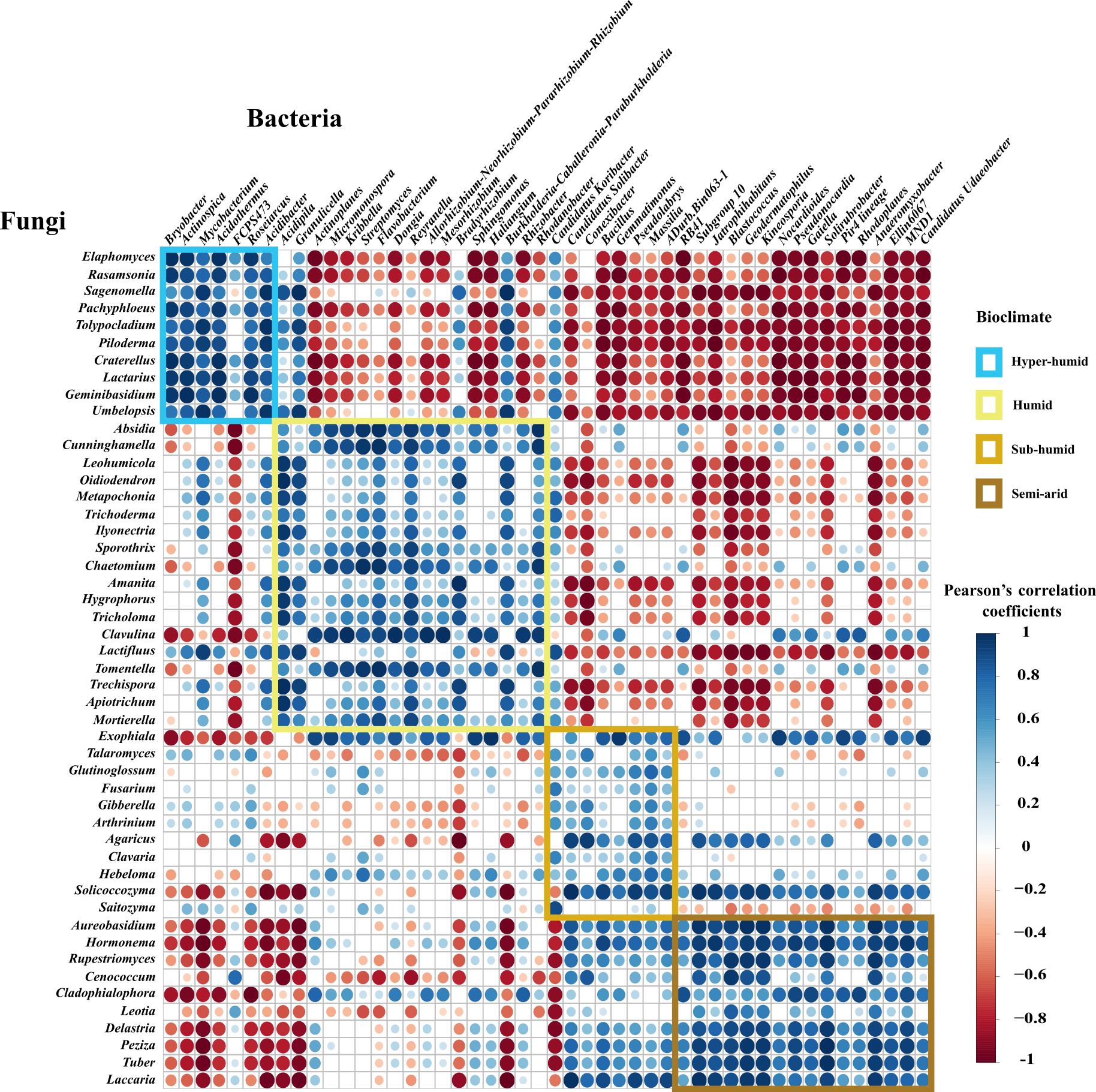


Figure S9. Correlation matrix between fungal and bacterial biomarkers of different bioclimates. Correlation matrix was computed using Pearson’s correlation coefficient with top 50 genus displayed in LefSe. Color intensity (from dark red to dark blue) ranges from negative (-1) to positive (1) Pearson's correlation coefficients (*p <*0.05). Circle size is proportional to the statistical significance. Blue rectangle contains biomarker genera of hyper-humid, yellow rectangle of humid, orange rectangle of sub-humid and brown rectangle of semi-arid bioclimate.
